# Electron-driven proton transfer relieves excited-state antiaromaticity in photoexcited DNA base pairs†

**DOI:** 10.1039/d0sc02294b

**Published:** 2020-08-12

**Authors:** Lucas J. Karas, Chia-Hua Wu, Henrik Ottosson, Judy I. Wu

**Affiliations:** Department of Chemistry, University of Houston Houston TX 77004 USA wujawa@gmail.com jiwu@central.uh.edu; Department of Chemistry, Ångström Laboratory, Uppsala University 751 20 Uppsala Sweden henrik.ottosson@kemi.uu.se

## Abstract

The Watson–Crick A·T and G·C base pairs are not only electronically complementary, but also photochemically complementary. Upon UV irradiation, DNA base pairs undergo efficient excited-state deactivation through electron driven proton transfer (EDPT), also known as proton-coupled electron transfer (PCET), at a rate too fast for other reactions to take place. Why this process occurs so efficiently is typically reasoned based on the oxidation and reduction potentials of the bases in their electronic ground states. Here, we show that the occurrence of EDPT can be traced to a reversal in the aromatic/antiaromatic character of the base upon photoexcitation. The Watson–Crick A·T and G·C base pairs are aromatic in the ground state, but the purines become highly antiaromatic and reactive in the first ^1^ππ* state, and transferring an electron and a proton to the pyrimidine relieves this excited-state antiaromaticity. Even though proton transfer proceeds along the coordinate of breaking a N–H σ-bond, the chromophore is the π-system of the base, and EDPT is driven by the strive to alleviate antiaromaticity in the π-system of the photoexcited base. The presence and absence of alternative excited-state EDPT routes in base pairs also can be explained by sudden changes in their aromatic and antiaromatic character upon photoexcitation.

## Abstract 2

The Watson–Crick A·T and G·C base pairs are not only electronically complementary, but also photochemically complementary.

## Graphical abstract

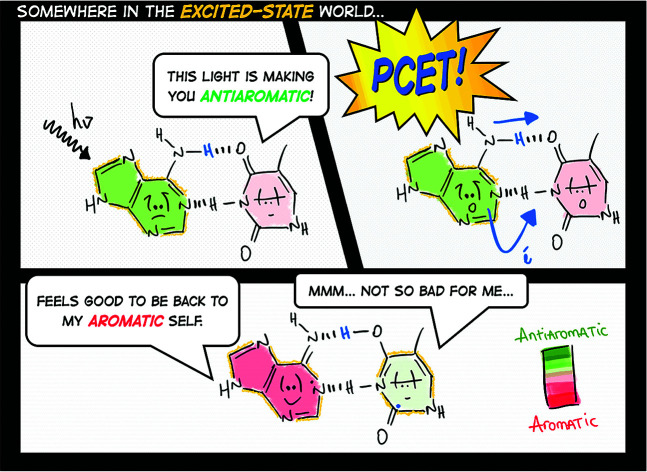

## Introduction

Before the development of an ozone layer in the Archean atmosphere, the flux of UV radiation reaching Earth was suggested to be several orders of magnitude higher than it is today. For the emerging biomolecules, constant exposure to strong UV irradiation meant that useful molecules had to be resistant to UV damage and harmful photochemical reactions. From this prebiotic environment, the Watson–Crick structures of A·T and G·C base pairs survived to encode genetic information—and the photostability of these winning pairs in this specific arrangement is astonishing.^1–3^ Upon UV irradiation, the photoexcited canonical base pairs undergo electron-driven proton transfer (EDPT), also labelled as proton coupled electron transfer (PCET), followed by non-radiative decay, and convert internally to the electronic ground state within picoseconds.^3–7^ Non-canonical conformers of A·T and G·C have been shown to display much longer excited-state lifetimes.^8^ We wish to suggest a reasoning for the special photostability of Watson–Crick A·T and G·C base pairs, based on the concepts of ground and excited-state aromaticity/antiaromaticity.

**Scheme 1:**
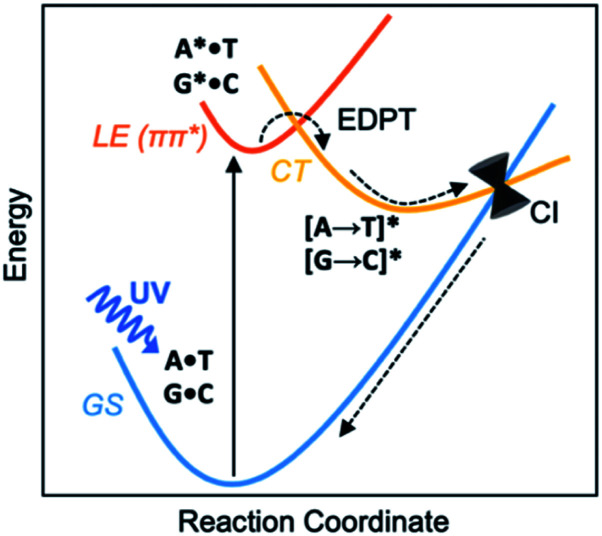
Excited-state deactivation in DNA base pairs.

It is understood that when isolated Watson–Crick structures of A·T and G·C are irradiated by short-wavelength light, they do not cross to a reactive triplet state, but convert internally to the electronic ground state through non-radiative decay—*i.e.*, *via* a “Domcke–Sobolewski channel”.^4–6,9,10^ Within picoseconds, the locally excited (LE) ^1^ππ* state connects to a charge-transfer (CT) state *via* EDPT, *i.e.*, an electron transfers from the purine (A or G) to the pyrimidine (T or C) and a proton follows. From there, the charge-transferred structure passes through a conical intersection (CI) and returns to the electronic ground state (GS) (Scheme 1). In this way, Watson–Crick base pairing reduces the excited state lifetimes of bases.^11,12^ Gas-phase experiments recorded short excited-state lifetimes for the isolated Watson-Crick structures of A·T (190 ps) and G·C (40 ps) base pairs.^13^ The rapid electron transfer reactions of photoexcited bases and base pairs are typically understood in terms of the early works of Rehm and Weller,^14,15^ showing a relationship between the rates of excited-state electron transfer reactions and the ground state oxidation and reduction potentials of the electron donating and accepting fragments. Excited-state lifetimes of base-stacked dinucleosides, for example, were found to correlate to the ionization potentials of the electron donating base minus the electron affinities of the electron accepting base.^16^

Here, we relate the efficient excited-state deactivation of isolated DNA base pairs to a sudden change in the aromatic and antiaromatic character of the electron donating and accepting bases. When irradiated by UV-light, the purines in the A·T and G·C base pairs become excited-state antiaromatic, and EDPT is the escape route to relieve antiaromaticity. This explanation complements the Rehm–Weller description for EDPT. Ionization potentials (and electron affinities) of compounds can be approximated by the negative of the energies of the highest occupied molecular orbital, HOMO, (and the lowest unoccupied molecular orbital, LUMO), and these quantities can be influenced by the aromatic (low HOMO energy, high LUMO energy) and antiaromatic (high HOMO energy, LUMO energy) character of the compounds. Yet, these properties are ground state properties. In contrast, our interpretation of EDPT considers the excited-state properties of paired bases, therefore supplements the Rehm–Weller explanation and enriches understanding of the process of photodeactivation of base pairs.

Hückel's 1931 paper^17^ first proposed a theory to determine the aromatic and antiaromatic characters of compounds using an electron-counting method. He suggested that closed-shell, cyclic, π-conjugated, organic compounds with [4*n* + 2] ring π-electrons exhibit aromatic character, and that those with [4*n*] ring π-electrons display antiaromatic character. On this basis, the purines (A and G, ten ring π-electrons) are aromatic and even the pyrimidines (T and C, six ring π-electrons) are weakly aromatic; the C=O π-bonds can be considered in their charge separated resonance forms C(*δ*+)–O(*δ*−) since the π-electrons are polarized towards the O atom. Baird suggested that these electron-counting rules reverse in the lowest excited ^3^ππ* states;^18^ compounds with [4*n*] ring π-electrons are aromatic and those with [4*n* + 2] ring π-electrons are antiaromatic. Later works found Baird's rule to extend also to the first ^1^ππ* states of organic compounds^19–22^ with significant interpretive merit for the photochemistry of organic compounds.^23^ In the ^1^ππ* state, the A, T, G, C bases are [4*n* + 2] π-antiaromatic. We now show that these features can explain important experimental observations of excited-state deactivation in isolated canonical and non-canonical A·T and G·C base pairs.

## Results and discussion

Potential energy curves for the ground state (GS), ^1^ππ* locally excited state (LE), and charge-transfer state (CT) of base pairs were computed at the CASPT2(8,8)/6-311+G(d,p)//(TD-)ωB97X-D/6-311+G(d,p) level with constrained *C*_s_ symmetry, employing Gaussian16 (ref. 24) for geometry optimization and Molpro2012.1 for single point calculations (see full methods in the ESI†).^25^ In the fully relaxed *C*_1_ geometries of A*·T and G*·C, the purine fragments are markedly puckered, but regain planarity in the CT states (see Fig. S7†); this suggests that the ^1^ππ* states of the purine fragments are excited-state antiaromatic, and distortion from planarity is one route to relieve antiaromatic character. Points on the LE and CT curves were computed by constraining the proton transferring N–H bonds to distances between 1 Å and 2.5 Å at varying increments of 0.1 Å. Points on the GS curves were computed based on single point energies of the corresponding optimized CT state geometries.

**Fig. 1:**
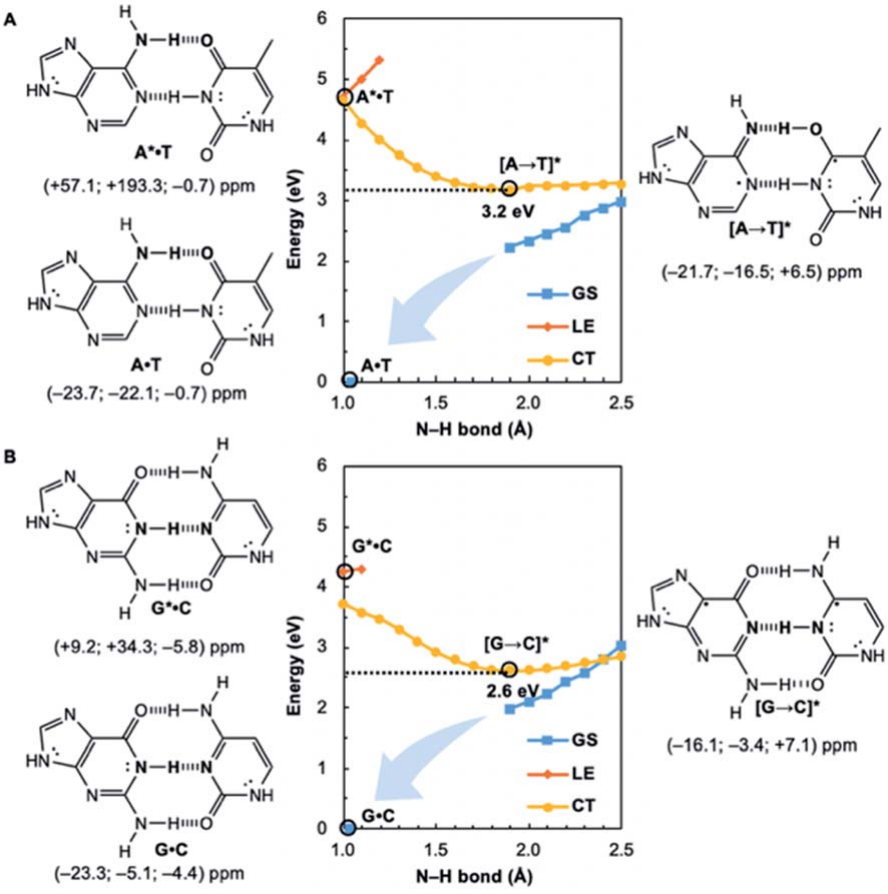
Potential energy functions of the electronic ground state (GS), ^1^ππ* locally-excited state (LE), and charge-transfer state (CT), with constrained N–H bond distance (see N–H in bold), for the Watson–Crick (A) A·T and (B) G·C structures, at CASPT2(8,8)/6-311+G(d,p). NICS(1)_*zz*_ values were computed at CASSCF(10,10)/6-311+G(d,p) for equilibrium structures at relevant positions on the potential energy curves (indicated by black circles).

**Fig. 2:**
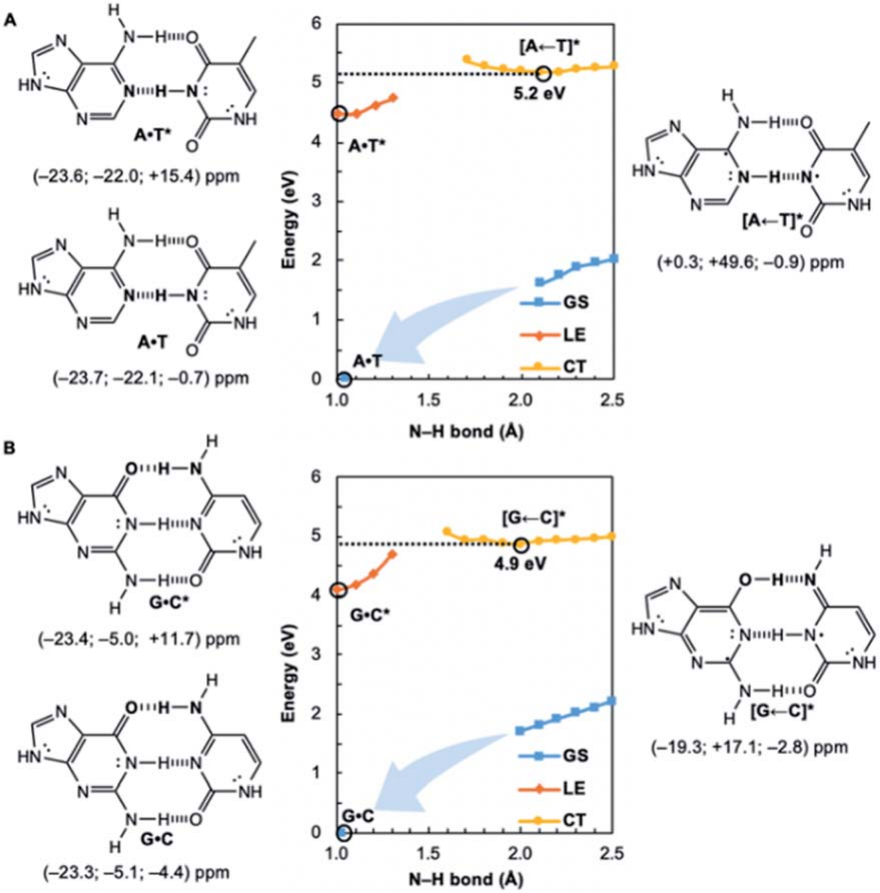
Potential energy functions of the electronic ground state (GS), locally-excited ^1^ππ* state (LE), and charge-transfer state (CT), with constrained N–H bond distance (see N–H in bold), for the Watson–Crick structures of (A) A·T and (B) G·C, with local excitations on pyrimidines, and computed NICS(1)_*zz*_ values for the equilibrium structures at relevant positions on the potential energy curves (indicated by black circles).

Nucleus independent chemical shifts, NICS(1)_*zz*_, were computed to quantify the aromatic and antiaromatic characters of base pair structures at relevant geometries (see black circles in Fig. 1, 2, and 4) on the GS, LE, and CT potential energy curves employing Dalton2016.^26^ NICS(1)_*zz*_ values are magnetic shielding tensors computed in the form of “ghost atoms” at 1 Å above ring centres, and reversed in sign to match experimental conventions for chemical shifts.^27–29^ Computed NICS at the ring centres of individual bases in base pairs were performed at the CASSCF(10,10)/6-311+G(d,p) level, based on base pair optimized geometries (see full methods in the ESI†). Negative NICS(1)_*zz*_ values indicate aromaticity (diatropicity), positive NICS(1)_*zz*_ values indicate antiaromaticity (paratropicity). Especially large positive NICS values can be an artefact of the NICS method and can occur for antiaromatic molecules with significant multiconfigurational character. Evaluations of aromaticity and antiaromaticity based on the multicenter index (MCI) method,^30^ an electronic index for aromaticity, are included in the ESI.†

Computed NICS(1)_*zz*_ for the Watson–Crick A·T and G·C base pairs show that the purines (A and G) are aromatic and the pyrimidines (T and C) are weakly aromatic in the electronic ground state (see Fig. 1). But upon photoexcitation, the purines become highly antiaromatic in the ^1^ππ* LE state; note large positive NICS(1)_*zz*_ for the A* (A*·T in Fig. 1A) and G* (G*·C in Fig. 1B) fragments. Crossing to the CT state relieves antiaromaticity. Following a barrierless EDPT reaction, the photoexcited purines lose an electron and a proton to the pyrimidines and regain aromatic character in the CT state. This re-aromatization stabilizes the CT state structures, [A → T]* (3.2 eV, relative to ground state A·T) and [G → C]* (2.6 eV, relative to ground state G·C). As a result, the S_1_ and S_0_ surfaces come close and a conical intersection is reached.

Notably, in A*·T and G*·C, proton transfer happens in the σ-framework, but the leaving electron must come from the π-framework. Even though proton transfer occurs along the coordinate of breaking a N–H σ-bond, the chromophore is the π-system of the base. A* and G* have negligible barriers to EDPT because a π-electron from the “excited-state antiaromatic” purine ring transfers to the 
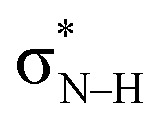
 orbital, and in effect an H atom leaves. Without involving transfer of a ring π-electron, homolytic cleavage of the N–H σ bond to give H· is highly endothermic, at both the ground and ^1^ππ* states of adenine and guanine (see energy plots of homolytic N–H σ-bond cleavage in Fig. S9†).

These findings have implications for the photostability of unnatural base pairs, which typically show electronic complementarity, but may not exhibit efficient excited-state deactivation pathways when irradiated by UV-light. Our results suggest that efficient EDPT reactions are most likely to occur when Hückel ground state aromatic bases are paired with non-aromatic bases. In this way, photoexcited base pairs that undergo EDPT reap the most benefit from excited-state antiaromaticity relief.

We wondered whether a similar mechanism could explain why EDPT in the reversed direction (*i.e.*, electron transfer and proton transfer from a photoexcited pyrimidine to the paired purine) is absent in Watson–Crick base pairs. Theoretical evidence has shown that EDPT cannot happen when excitation takes place on the pyrimidines of isolated canonical base pairs;^31,32^ crossing between the first ^1^ππ* state and the CT state involves a high barrier to EDPT. We found that when excitation occurs on the pyrimidines (T and C), they only become modestly antiaromatic (since these rings have breached ring π-electron delocalizations and are near-non-aromatic in the ground state), while the purines remain largely aromatic (see Fig. S8†).

Note the small positive NICS(1)_*zz*_ values for T* and C*, but negative NICS(1)_*zz*_ values for A and G, in the LE state structures of A·T* (Fig. 2A) and G·C* (Fig. 2B). When and if EDPT happens, the purported equilibrium CT state structures, [A ← T]* (5.2 eV relative to ground state A·T) and [G ← C]* (4.9 eV relative to ground state G·C), are relatively high in energy since the purines gain an electron (11 ring π-electrons) and lose aromatic character (Fig. 2). EDPT is disfavored, since there is less drive to relieve antiaromaticity in the LE state, and the CT state structure is not especially stabilized by aromatic character in the purines or pyrimidines. This may explain why an EDPT deactivation route is not viable for the locally excited Watson–Crick structures of A·T* and G·C*, why these reactive states must deactivate through other pathways,^13^ and possibly why DNA base pair damage often takes place on the pyrimidines, like thymine.^7^

**Fig. 3:**
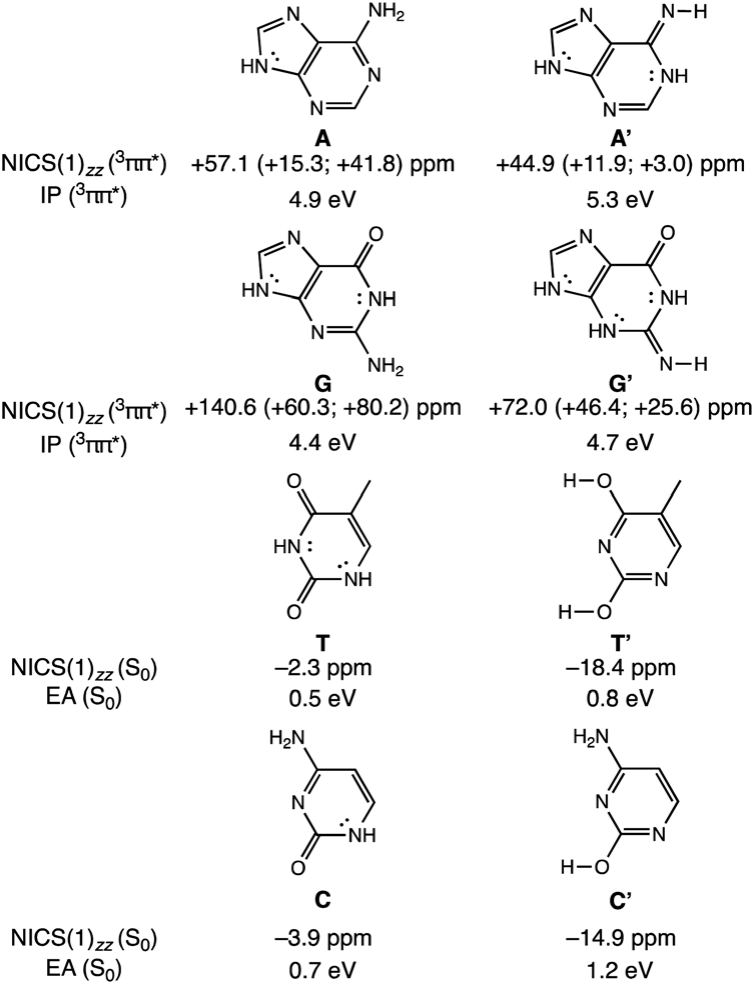
Computed vertical ionization potentials (IP) in the ^3^ππ* states of purines (A and G) and vertical electron affinities in the ground states of pyrimidines (T and C), compared to their isomers (A′, G′, T′, C′) at ωB97X-D/6-311+G(d,p). Dissected NICS(1)_*zz*_ values were computed at PW91/IGLOIII.

To clarify how our explanation based on the relief of excited-state antiaromaticity is connected with the established Rehm–Weller model, we considered the ionization energy of the photoexcited bases and the electron affinities of their hydrogen-bonding partners. As a model, we investigated the ^3^ππ* triplet state ionization potentials (IP) of the purines and their 1,3-hydrogen shifted (non-aromatic) isomers, as well as the ground state electron affinities (EA) of the pyrimidines relative to comparable (aromatic) isomers (Fig. 3). Noteworthy, the original Rehm–Weller model is based on the IPs of the ground state, yet, a more elaborate approach is based on the excited state IPs, as considered by Stanley and co-workers in analogues of adenine.^33^ While the IPs of compounds reflect their potentials to become oxidized, and the EAs reflect the potential to reduce, these energetic quantities are largely affected by structural variations, for example, based on differences in functional groups, heteroatoms, and π-conjugation patterns such as aromatic and antiaromatic character.

A comparison between the computed vertical ^3^ππ* state IP of adenine (A) (4.9 eV) and its isomer A′ (5.3 eV, Fig. 3) now shows that the IP for the ^3^ππ* state of A (with cyclic [4*n* + 2] π-delocalization) is lower by 0.4 eV than that of A′ (breached [4*n* + 2] π-delocalization, due to an exocyclic imine). This could be attributed to a more pronounced excited-state antiaromatic character of A than of A′ (see NICS(1)_*zz*_ values, Fig. 3), making removal of an electron from A easier. Similarly, the computed vertical ^3^ππ* state IP of guanine (G) (4.4 eV) is lower by 0.3 eV than that of its isomer G′ (4.7 eV) and can be explained by the same rationale (Fig. 3). Even though both G and G′ are formally [4*n* + 2] π-conjugated, cyclic delocalization is breached by one exocyclic group in G (one C=O group) but by two in G′ (one C=O and one C=NH group). Consequently, the ^3^ππ* state of G is more antiaromatic than that of G′ (see NICS(1)_*zz*_ values, Fig. 3), and removing an electron from G is easier.

The connection of excited state IPs to excited state antiaromaticity in [4*n* + 2] π-electron compounds and excited state aromaticity in [4*n*] π-electron compounds is further corroborated through computations of the IPs of benzene, pyrrole, cyclobutadiene and borole in their lowest ^3^ππ* states.^34^ As measured by EAs, the tendency of pyrimidines to receive an electron, can be related to their ground state aromatic character. As shown in Fig. 3, T (0.5 eV) and C (0.7 eV) show a lower vertical EA compared to the more aromatic isomers, T′ (0.8 eV) and C′ (1.2 eV). See also computed triplet state IPs for the pyrimidines and ground state EAs for the purines in Fig. S10.†

In this way, the power to be photo-oxidized (based on the triplet state IPs) and the power to be reduced (based on the ground state EAs), can be related to, respectively, the excited-state antiaromatic character of the purines and the weak ground state aromatic character of the pyrimidines. Furthermore, it has been shown that the aromatic and antiaromatic character of compounds have direct consequence for their energetic stabilization and destabilization in the ground^35^ and excited states.^36^ In the triplet state, compounds with [4*n* + 2] π-antiaromatic character are destabilized while those with [4*n*] π-aromatic character are stabilized, when compared to their nonaromatic isomers.^36^ Therefore, explanations of the efficient photodeactivation of base pairs, based on either ionization potentials/electron affinities (Rehm–Weller model) or changes in (anti)aromaticity are two sides of the same coin, yet, the excited state antiaromaticity based model clarifies why the IPs of the photoexcited purines are low.

**Fig. 4:**
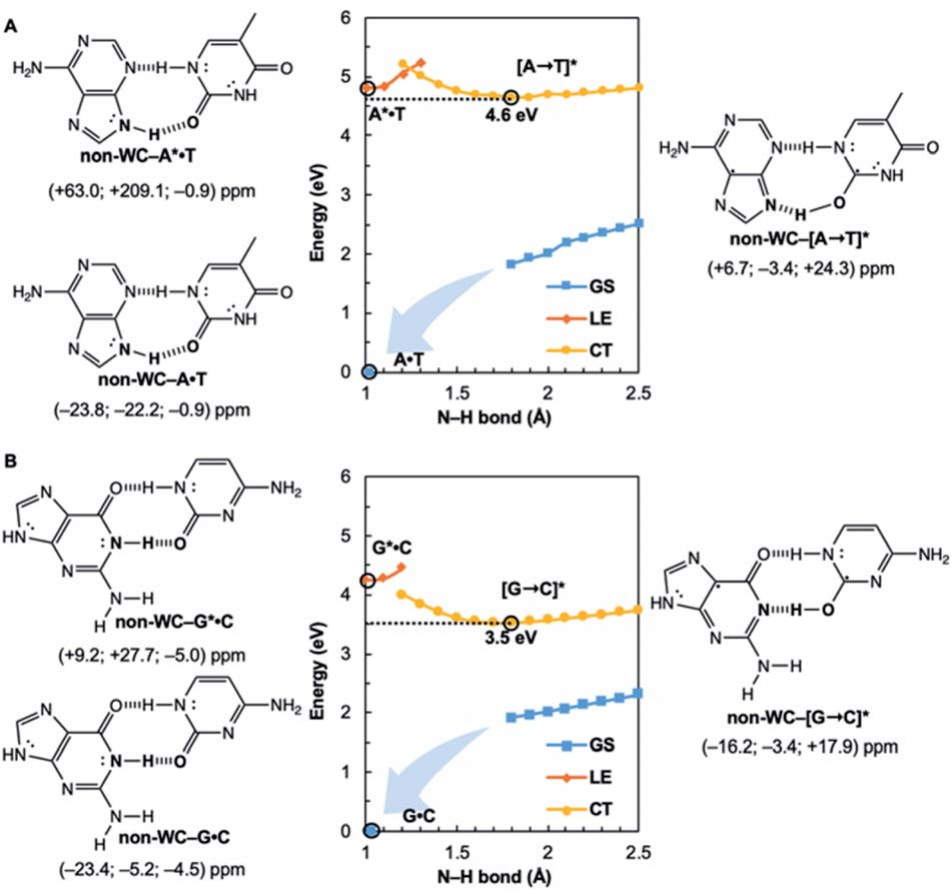
Potential energy functions of the electronic ground state (GS), locally-excited ^1^ππ* state (LE), and charge-transfer state (CT), with constrained N–H bond distance, for the most stable non-Watson–Crick (non-WC) forms of (A) A·T and (B) G·C, and computed NICS(1)_*zz*_ values for the equilibrium structures at relevant positions on the potential energy curves (indicated by black circles).

Are the Watson–Crick arrangements special? Experiments have found the isolated Watson–Crick G·C pair to exhibit a broad UV peak, compared to sharp peaks for alternative arrangements, and suggesting a short excited-state lifetime.^8^ IR-UV spectra for several isomers of isolated A·T pairs were found to match well with computed IR spectra of the supposed structures.^37^ But that of the Watson–Crick pair matched poorly (resembling the spectra for a Hoogsteen pair instead), possibly due to a short excited-state lifetime. Computational evidence showed that many isolated non-canonical A·T and G·C base pair arrangements lacked the required conical intersection for rapid excited-state deactivation.^5,6^ We considered the most stable non-Watson–Crick (non-WC) conformers of A·T^37^ and G·C^5^ and found the purines to be antiaromatic in the ^1^ππ* LE states—just like the Watson–Crick forms (Fig. 4). But when EDPT happens, the equilibrium CT state structures, non-WC–[A → T]* and non-WC–[G → C]*, are stabilized by aromaticity to a lesser extent; the purine rings show only weak to modest aromatic character, but the pyrimidines are antiaromatic. As a result, these non-canonical CT state structures are higher in energy: non-WC–[A → T]* (4.6 eV relative to ground state non-WC–A·T) and non-WC–[G → C]* (3.5 eV relative to ground state non-WC–G·C) (*cf.* relative energy of CT structures in Fig. 1), and a conical intersection is less likely to be reached.

Gauge including magnetically induced current (GIMIC) plots were computed at B3LYP/6-311+G(d,p) using base pair geometries in the GS, and agree with the computed NICS(1)_*zz*_ results. Plots for the GS states were modelled by a summation of ring currents computed for the purine (S_0_ state) and the pyrimidine (S_0_ state) fragments. Plots for the LE states were modelled by a summation of ring currents computed for the purine (T_1_ state) and the pyrimidine (S_0_ state) fragments. Plots for the CT states were modelled by a summation of ring currents computed for the purine (D_0_ state) and the pyrimidine (D_0_ state) fragments.

**Fig. 5:**
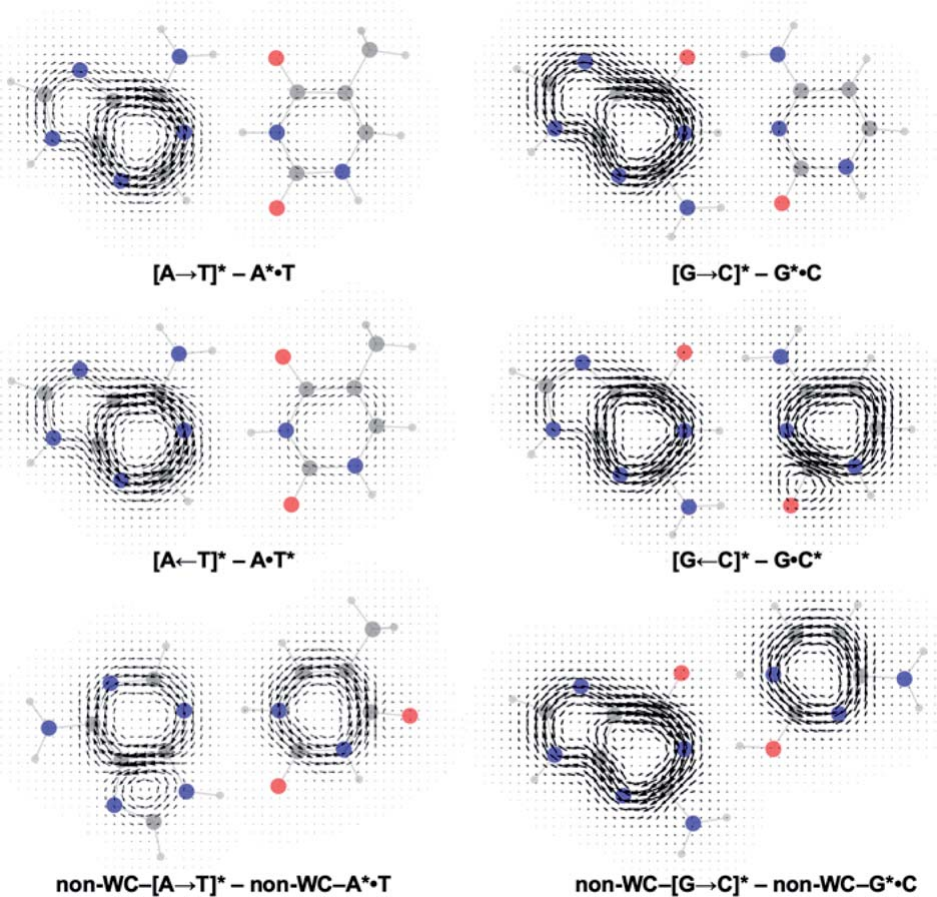
Computed ΔGIMIC ring current plots for CT–LE states of the Watson–Crick A·T pair and G·C pairs.

ΔGIMIC plots for the CT minus LE states are shown in Fig. 5 (see original GIMIC plots for all GS, LE, and CT states in Fig. S5†). When excitation occurs on the purine, the Watson–Crick (top row) and non-Watson–Crick (bottom row) pairs display increased diatropicity (clockwise current, due to antiaromaticity relief) on the purine fragments upon charge transfer to the CT states. Note stronger ΔGIMIC current intensity for the Watson–Crick pairs compared to the non-Watson–Crick pairs. ΔGIMIC currents for the pyrimidine fragments show increased paratropicity (anticlockwise current) and are weaker in intensity for the Watson–Crick A·T and G·C pairs compared to the non-Watson–Crick pairs. When excitation occurs on the pyrimidine (middle row), charge transfer from the pyrimidine to the purine increases paratropicity (anticlockwise current, suggesting increased antiaromatic character) on the purine fragment, and the pyrimidine fragments show weak to moderate increase in diatropicity (clockwise current).

Besides NICS(1)_*zz*_,^38,39^ computed multicenter index (MCI) (Tables S8 and S9†), harmonic oscillator model of electron delocalization (HOMED)^40^ (Table S7†), and computed H NMR shifts (Table S6†) also support the reported findings. Even though the MCI and HOMED methods were not designed to capture the effects of excited-state (anti)aromaticity, results based on these methods suggest that changes in (anti)aromatic character are relevant for interpreting the EDPT mechanism of base pairs.

**Scheme 2:**
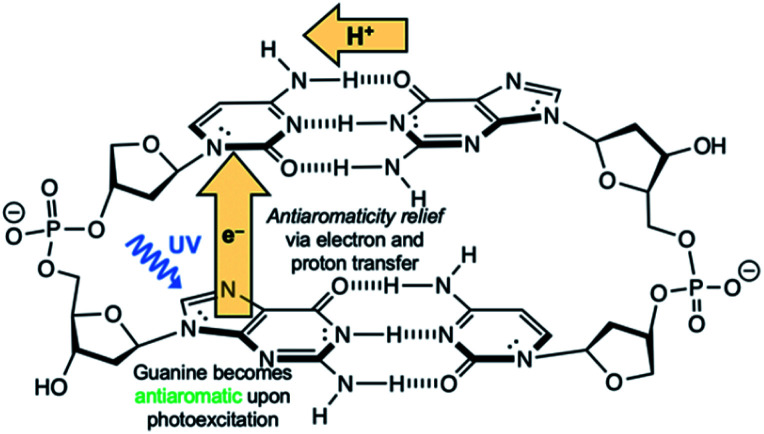
Schematic illustration EDPT in stacked G·C:C·G DNA duplex.

Another competitive EDPT pathway of double-stranded DNA involves intra-strand electron transfer between stacked nucleobases, followed by inter-strand proton transfer in the resulting radical anion base pair.^41,42^ But the effects of excited-state antiaromaticity described here for explaining photodeactivation in isolated base pairs may very well apply. When a purine base becomes antiaromatic in the photoexcited state, an electron has to leave to relieve excited-state antiaromaticity, but it can depart through inter-strand electron transfer or intra-strand electron transfer. For example, in the G·C:C·G DNA duplex (Scheme 2), a photoexcited guanine can transfer an electron through intra-strand stacking to a neighbouring cytosine to relieve excited-state antiaromaticity (see Fig. S11 and Table S11 in the ESI†), and proton transfer involves a different guanine fragment. In this proposed EDPT pathway,^41,42^ separate purine fragments participate in electron transfer and proton transfer to a pyrimidine, but the net result is the same.

## Conclusions

It is tempting to imagine that textbook concepts like aromaticity and antiaromaticity may have played a decisive role in the molecular evolution of privileged bases and base pairs towards encoding genetic information. When DNA base pairs are irradiated by light, the purines become excited-state antiaromatic, and to relieve excited-state antiaromaticity an electron must go away—either by departing to its hydrogen bonded pair or to a neighbouring stacked base, and proton transfer follows. Whether or not EDPT happens is determined by changes in the π-electronic structure of the photoexcited base. These findings have immediate implications for the design of photostable unnatural base pairs, as well as other light-driven proton transfer^43^ and electron transfer processes.

The concept of excited-state aromaticity and antiaromaticity is being applied to a growing number of areas in chemistry, such as synthetic method developments,^44,45^ as well as the design of light-active molecules and materials,^46–49^ fluorophores,^50^ and materials for energy conversion.^51,52^ We suggest that this concept also has relevance for understanding one of the most fundamental photochemical processes in biochemistry.

## Conflicts of interest

There are no conflicts to declare.

## Supplementary Material

SC-011-D0SC02294B-s001
