# Supplementary material for: Electron-driven proton transfer relieves excited-state antiaromaticity in photoexcited DNA base pairs
Source: Chem Sci. 2020 Aug 12;11(37):10071–7. doi: 10.1039/d0sc02294b (PMC8162126; doi:10.1039/d0sc02294b)
Supplement: SC-011-D0SC02294B-s001 [file SC-011-D0SC02294B-s001.pdf]

Electronic Supplementary Material (ESI) for Chemical Science.
This journal is © The Royal Society of Chemistry 2020

Electron-Driven Proton Transfer Relieves Excited-State Antiaromaticity in Photoexcited DNA Base Pairs

Lucas J. Karas,[a] Chia-Hua Wu,[a]* Henrik Ottosson,[b]* Judy I. Wu[a]*

[a]Department of Chemistry, University of Houston, Houston, TX 77004, USA

[b]Department of Chemistry, Ångström Laboratory, Uppsala University, 751 20, Uppsala, Sweden

Correspondence: wujawa@gmail.com; henrik.ottosson@kemi.uu.se; jiwu@central.uh.edu

Table of Contents

1. Computational Methods. 4

2. Computed electron density difference (EDD) maps 5

Figure S1. Electron density (ρ) difference maps showing local excitations [ρ(LE) – ρ(GS)] (left) and charge transfer [ρ(CT) – ρ(GS)] (right) in the: A) Watson–Crick (WC) A•T base pair, B) pyrimidine excitation in the WC A•T base pair, and C) non-Watson–Crick (non-WC) A•T base pair. EDD maps were computed at CASSCF(8,8)/6-311+G(d,p) using equilibrium LE and CT state geometries. 5

Figure S2. Electron density (ρ) difference maps showing local excitations [ρ(LE) – ρ(GS)] (left) and charge transfer [ρ(CT) – ρ(GS)] (right) in the: A) Watson–Crick (WC) G•C base pair), B) pyrimidine excitation in the WC G•C base pair, C) non-Watson–Crick (non-WC) G•C base pair, and D) alternative proton transfer pathway in the WC G•C base pair. EDD maps were computed at CASSCF(8,8)/6-311+G(d,p) using equilibrium LE and CT state geometries. 6

3. Computed NICS(1)$_{zz}$ values for base fragments in base pair structures vs. base pairs 7

Table S1. Computed NICS(1)$_{zz}$ for base fragments in base pair structures vs. base pairs, in the ground state, at PW91/IGLOIII. 7

4. Computed carbon shieldings at CASSCF/6-311+G(d,p), with different active spaces. 8

Figure S3. Carbon numbering for adenine (A), thymine (T), guanine (G), and cytosine (C). 8

Table S2. Computed carbon shieldings in the adenine (A) fragment (based on WC A•T geometry) at the CASSCF/6-311+G(d,p) level with different active space sizes. 8

Table S3. Computed carbon shieldings in the thymine (T) fragment (based on WC A•T geometry) at the CASSCF/6-311+G(d,p) level with different active space sizes. 8

Table S4. Computed carbon shieldings in the guanine (G) fragment (based on WC G•C geometry) at the CASSCF/6-311+G(d,p) level with different active space sizes. 8

Table S5. Computed carbon shieldings in the cytosine (C) fragment (based on WC G•C geometry) at the CASSCF/6-311+G(d,p) level with different active space sizes. 9

5. Computed ^{1}H chemical shifts. 10

Figure S4. Hydrogen numbering for adenine (A), thymine (T), guanine (G), and cytosine (C). 10

Table S6. Computed ^{1}H chemical shift in ppm for the Watson-Crick and non-Watson-Crick of A•T and G•C in different electronic states at the CASSCF(10,10)/6-311+G(d,p) level. 10

6. Computed GIMIC ring current plots 11

Figure S5. Computed GIMIC ring current plots for the GS (left column), LE (middle column), and CT (right column) states of the: A) Watson–Crick A•T and G•C pairs (excitation on purines), B) Watson–Crick A•T and G•C pairs (excitation on the pyrimidines), and C) non-Watson–Crick A•T and G•C pairs (excitation on purines). 11

7. Computed Harmonic Oscillator Model of Electron Delocalization (HOMED) 12

Table S7. *HOMED values calculated at ωB97XD/6-311+G(d,p) for the Watson-Crick and non-Watson-Crick forms of A•T and G•C in different electronic states.* 12

8. Multicenter indices (MCI) values 13

Table S8. *Computed MCI values at the CASSCF(8,8)/6-311+G(d,p) level for A•T.* 13
Table S9. *Computed MCI values at the CASSCF(8,8)/6-311+G(d,p) level for G•C.* 13

9. Computed MCI values for Watson–Crick A•T and G•C structures at C_1 symmetry 14

Figure S6. *Energies and MCI values based on minima geometries of the GS, LE, and CT states, for: A) WC A•T, and B) WC G•C, at (TD-)ωB97XD/6-311+G(d,p).* 14
Figure S7. *Minima geometries for the LE and CT states of: A) WC A•T and B) WC G•C.* 15

10. Alternative proton-coupled electron transfer pathway in the Watson–Crick G•C base pair. 16

Figure S8. *Potential energy functions of the electronic ground state (GS), locally-excited $^1\pi\pi^*$ state (LE), and charge-transfer state (CT), with constrained N–H bond distance, for the Watson-Crick structure of G•C involving the exocyclic NH_2 of guanine at CASPT2(8,8)/6-311+G(d,p). NICS(1)$_{zz}$ values were computed for relevant structures at the CASSCF(10,10)/6-311+G(d,p) level.* 16

11. Homolytic N–H σ-bond cleavage of adenine and guanine. 17

Figure S9. *Potential energy functions for constrained N–H stretching (see N–H bond in bold) of the ground state (GS) and locally-excited $^1\pi\pi^*$ state (LE) of A) adenine and B) guanine at CASPT2(10,10)/6-311+G(d,p).* 17

12. Computed vertical ionization potentials (IP) and electron affinities (EA) 18

Figure S10. *Computed vertical ionization potentials (IP) in the $^3\pi\pi^*$ states of pyrimidines (**T** and **C**) and vertical electron affinities (EA) in the ground states of purines (**A** and **G**), compared to their isomers (**A'**, **G'**, **T'**, **C'**) at ωB97X-D/6-311+G(d,p). Dissected NICS(1)$_{zz}$ values were computed at PW91/IGLOIII.* 18

13. DNA duplex model study 19

Figure S11. *Proton-coupled electron transfer in the G•C:C•G DNA duplex.* 19
Figure S12. *Optimized structure of guanine **1** in the LE state and CT state. Note that **1** is non-planar in the LE state, but becomes planar upon electron transfer to cytosine **3**.* 19
Table S10. *Relative energies (in eV) for the ground state, locally-excited (LE) state, charge transfer (CT) state, and proton-coupled electron transfer state (PCET) of the G•C:C•G duplex at (TD-)ωB97XD/6-31+G(d)//ONIOM(ωB97XD/6-31+G(d):PM6).* 20
Table S11. *Computed multicenter index (MCI) values for each ring system, at (TD-)ωB97XD/6-31+G(d)//ONIOM (ωB97XD/6-31+G(d):PM6).* 20

14. References for Software and Programs 21

15. CASPT2(8,8)/6-311+G(d,p) energies for point on the potential energy curves. 22

Table S12. *Computed total electronic energies (in Hartree) for the GS, LE, and CT states of WC A•T, as a function of N–H bond stretching (see bold-type N–H bond in Figure 2A).* 22
Table S13. *Computed total electronic energies (in Hartree) for the GS, LE, and CT states of WC A•T (excitation on pyrimidine), as a function of N–H bond stretching (see bold-type N–H bond in Figure 3A).* 22
Table S14. *Computed total electronic energies (in Hartree) for the GS, LE, and CT states of non-WC A•T, as a function of N–H bond stretching (see bold-type N–H bond in Figure 4A).* 23
Table S15. *Computed total electronic energies (in Hartree) for the GS, LE, and CT states of WC G•C, as a function of N–H bond stretching (see bold-type N–H bond in Figure 2B).* 23

Table S16. *Computed total electronic energies (in Hartree) for the GS, LE, and CT states of WC G•C (excitation on pyrimidine), as a function of N–H bond stretching (see bold-type N–H bond in Figure 3B).*...................................24
Table S17. *Computed total electronic energies (in Hartree) for the GS, LE, and CT states of non-WC G•C, as a function of N–H bond stretching (see bold-type N–H bond in Figure 4B).* 24
Table S18. *Computed total electronic energies (in Hartree) for the GS, LE, and CT states of WC G•C (alternative PCET pathway), as a function of N–H bond stretching (see bold-type N–H bond in Figure S6).* 25

16. Optimized Cartesian coordinates for relevant structures...**26**

1. Computational Methods.

Equilibrium geometries for all base pairs, in the ground state (GS), locally-excited state (LE), and charge transfer state (CT), were optimized with C_s symmetry at (TD-)ωB97XD/6-311+G(d,p) employing Gaussian16. Potential energy curves for the LE and CT states were constructed based on constrained geometry optimizations, in which selected N–H bonds were set to distances between 1Å and 2.5 Å, and varied at increments of 0.1 Å. Ground state potential energy curves were computed based on single point energies of the corresponding optimized CT state geometries. All energy points on the GS, LE, and CT curves were computed at CASPT2(8,8)/6-311+G(d,p) using an active space of eight orbitals (four occupied and four unoccupied π-orbitals, with eight electrons) employing Molpro2012.1. Electron density difference maps verified local excitations or charge-transfers for relevant structures (Figures S1 and S2).

Based on the equilibrium geometries of base pairs, dissected nucleus-independent chemical shifts, NICS(1)$_{zz}$, were computed for each base, by splitting the optimized structures of base pairs into individual bases and considering each fragment separately (with the purported charge and electronic state). NICS(1)$_{zz}$ values were computed at 1 Å above the five and six membered ring centers and include only contributions from the "out-of-plane" (zz) tensor component perpendicular to the ring plane. All NICS(1)$_{zz}$ data were computed at CASSCF(10,10)/6-311+G(d,p) using an active space of ten orbitals (five occupied and five unoccupied π-orbitals, with ten electrons), employing the Dalton2016 program. Choice of active space for NICS(1)$_{zz}$ calculations was selected by comparing computed carbon shieldings at the CASSCF/6-311+G(d,p) level with different active space sizes (Tables S2, S3, S4 and S5). Computed NICS(1)$_{zz}$ at the PW91/IGLOIII level for separate bases in base pair structures were benchmarked against results for the corresponding base pairs and showed excellent agreement (Table S1). Multicenter indices (MCI) values were computed for base pairs structures at CASSCF(8,8)/6-311+G(d,p) using an active space of eight orbitals (four occupied π orbitals and four unoccupied π-orbitals, with eight electrons) employing ESI-3D.

2. Computed electron density difference (EDD) maps

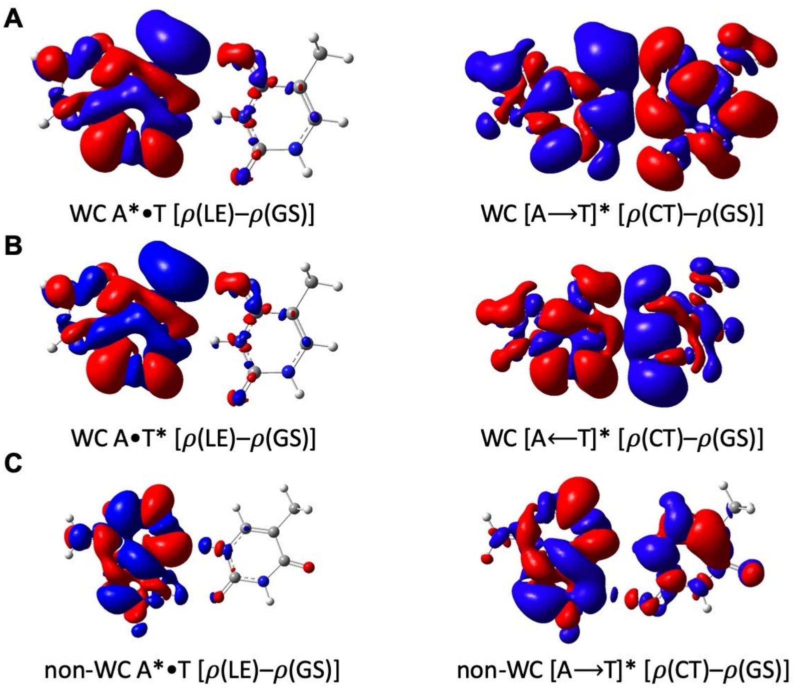

Figure S1. Electron density (ρ) difference maps showing local excitations [ρ(LE) – ρ(GS)] (left) and charge transfer [ρ(CT) – ρ(GS)] (right) in the: A) Watson–Crick (WC) A•T base pair, B) pyrimidine excitation in the WC A•T base pair, and C) non-Watson–Crick (non-WC) A•T base pair. EDD maps were computed at CASSCF(8,8)/6-311+G(d,p) using equilibrium LE and CT state geometries.

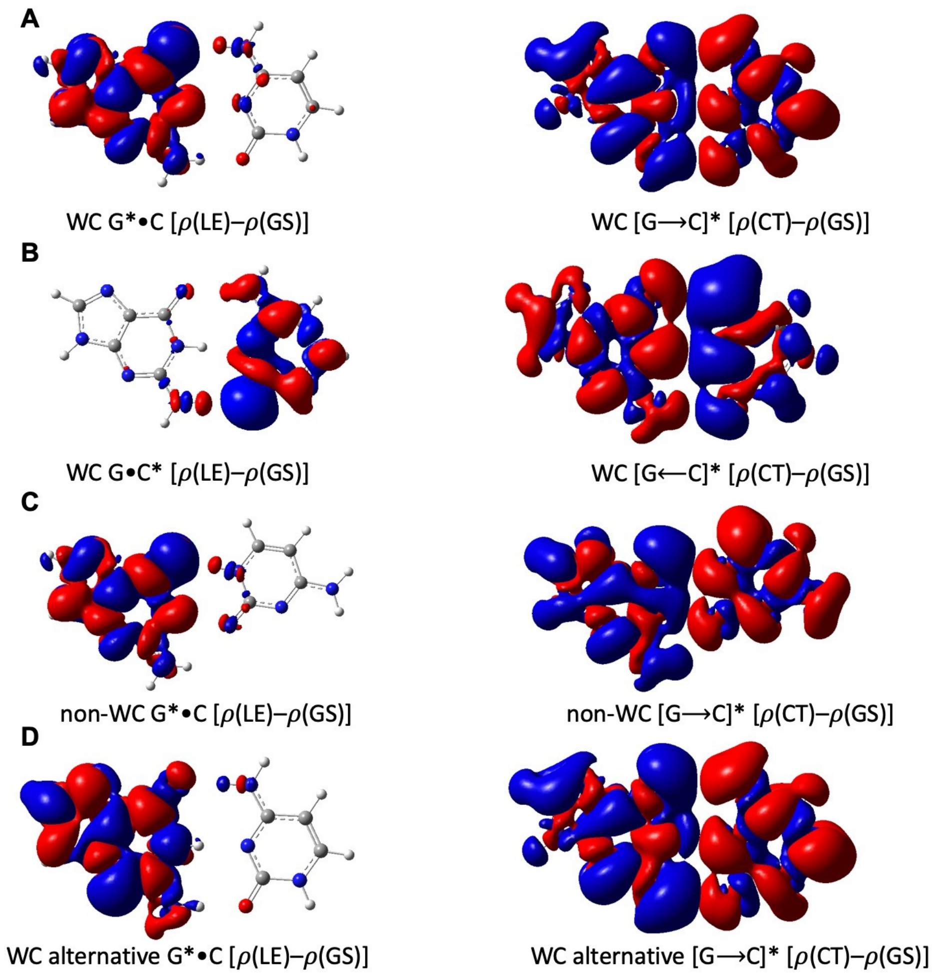

Figure S2. Electron density (ρ) difference maps showing local excitations [ρ(LE) – ρ(GS)] (left) and charge transfer [ρ(CT) – ρ(GS)] (right) in the: A) Watson–Crick (WC) G•C base pair), B) pyrimidine excitation in the WC G•C base pair, C) non-Watson–Crick (non-WC) G•C base pair, and D) alternative proton transfer pathway in the WC G•C base pair. EDD maps were computed at CASSCF(8,8)/6-311+G(d,p) using equilibrium LE and CT state geometries.

3. Computed NICS(1)$_{zz}$ values for base fragments in base pair structures vs. base pairs

Table S1. Computed NICS(1)$_{zz}$ for base fragments in base pair structures vs. base pairs, in the ground state, at PW91/IGLOIII.

		Purine (6MR)	Purine (5MR)	Pyrimidine
WC A•T	base fragment[a]	-20.4	-26.7	-2.2
	base pair	-17.8	-26.2	-2.0
non-WC A•T	base fragment[a]	-20.4	-26.7	-2.2
	base pair	-19.2	-27.2	-2.5
WC G•C	base fragment[a]	-5.9	-24.0	-3.9
	base pair	-6.2	-23.2	-2.9
non-WC G•C	base fragment[a]	-5.9	-24.1	-3.8
	base pair	-7.0	-23.6	-6.1

[a]NICS(1)$_{zz}$ calculated by splitting the optimized structures of base pairs into two bases and considering each fragment separately.

4. Computed carbon shieldings at CASSCF/6-311+G(d,p), with different active spaces.

Based on ground state equilibrium structures of the WC A•T and G•C base pairs, carbon shieldings for the base fragments were computed to identify an appropriate active space for NICS calculations at the CASSCF level. Base fragments were considered so that a larger active space (up to 12,12) could be examined with the 6-311+G(d,p) basis set. Computed carbon shielding values converged at an active space size of 10 π-orbitals (five occupied and five unoccupied π-orbitals) and 10 electrons (10,10).

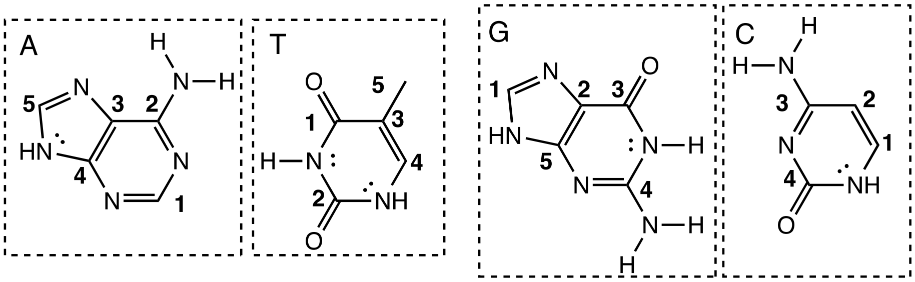

Figure S3. Carbon numbering for adenine (A), thymine (T), guanine (G), and cytosine (C).

Table S2. Computed carbon shieldings in the adenine (A) fragment (based on WC A•T geometry) at the CASSCF/6-311+G(d,p) level with different active space sizes.

	Active space size (π orbitals only)					
Atom / Shielding (ppm)	(2,2)	(4,4)	(6,6)	(8,8)	(10,10)	(12,12)
C_1	26.5	46.7	45.4	49.6	49.5	49.6
C_2	27.1	36.7	35.6	42.7	44.5	44.4
C_3	81.9	74.3	78.6	82.7	83.1	82.7
C_4	30.9	40.5	37.6	48.4	48.4	50.8
C_5	65.2	48.9	64.0	63.1	63.4	66.3

Table S3. Computed carbon shieldings in the thymine (T) fragment (based on WC A•T geometry) at the CASSCF/6-311+G(d,p) level with different active space sizes.

	Active space size (π orbitals only)					
Atom / Shielding (ppm)	(2,2)	(4,4)	(6,6)	(8,8)	(10,10)	(12,12)
C_1	19.2	35.6	35.7	35.7	37.1	37.1
C_2	35.8	35.9	37.5	48.6	49.9	50.7
C_3	92.5	93.9	93.5	94.8	94.5	93.8
C_4	70.9	72.9	73.6	73.4	73.5	73.5
C_5	181.2	181.3	181.3	181.3	181.3	181.1

Table S4. Computed carbon shieldings in the guanine (G) fragment (based on WC G•C geometry) at the CASSCF/6-311+G(d,p) level with different active space sizes.

	Active space size (π orbitals only)					
Atom / Shielding (ppm)	(2,2)	(4,4)	(6,6)	(8,8)	(10,10)	(12,12)
C_1	69.2	67.8	70.3	66.8	67.3	70.3
C_2	82.6	85.9	85.0	83.0	83.4	83.6
C_3	27.0	25.6	25.6	28.1	42.1	40.8
C_4	30.6	31.9	31.9	46.8	46.6	45.5

C_5	30.5	44.2	47.5	48.7	50.3	52.4

Table S5. Computed carbon shieldings in the cytosine (C) fragment (based on WC G•C geometry) at the CASSCF/6-311+G(d,p) level with different active space sizes.

	Active space size (π orbitals only)				
Atom / Shielding (ppm)	(2,2)	(4,4)	(6,6)	(8,8)	(10,10)
C_1	59.9	63.3	64.8	65.1	65.2
C_2	110.2	109.8	109.6	110.9	111.2
C_3	16.6	29.1	30.7	32.3	34.5
C_4	30.9	30.5	33.0	44.7	44.8

5. Computed ^{1}H chemical shifts.

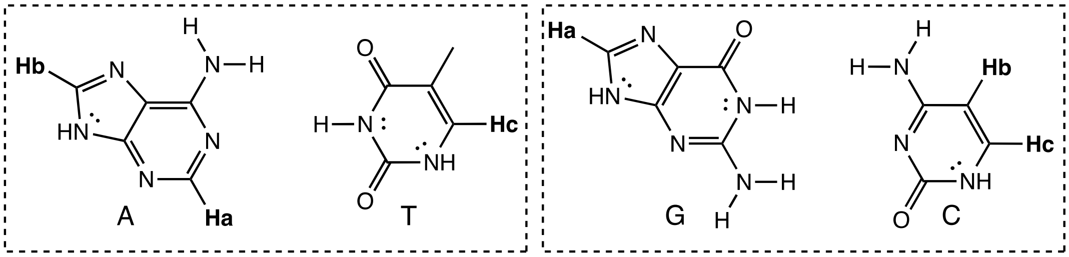

Figure S4. Hydrogen numbering for adenine (A), thymine (T), guanine (G), and cytosine (C).

Table S6. Computed ^{1}H chemical shift in ppm for the Watson-Crick and non-Watson-Crick of A•T and G•C in different electronic states at the CASSCF(10,10)/6-311+G(d,p) level.

Base Pair Structure	Ha	Hb	Hc
A•T	7.3	8.2	6.0
A*•T	1.0	3.0	6.0
[A→T]*	8.7	7.4	4.4
G•C	6.9	4.4	6.4
G*•C	5.7	4.8	6.8
[G→C]*	7.3	4.2	4.4
A•T*	8.1	7.3	5.5
[A←T]*	3.7	5.6	6.2
G•C*	6.9	4.6	4.3
[G←C]*	6.5	4.7	6.3
non-WC A•T	8.1	7.2	5.9
non-WC A*•T	0.5	2.6	5.9
non-WC [A→T]*	7.8	7.8	3.4
non-WC G•C	6.9	6.8	6.4
non-WC G*•C	6.1	4.6	6.4
non-WC [G→C]*	7.3	4.1	4.1

The base pair ^{1}H chemical shifts were calculated using the following equation.

$$\delta_{BP} = \sigma_{benz} - \sigma_{BP} + \delta_{benz}$$

σ_{BP} is the calculated shielding of the base pairs, σ_{benz} is the calculated shielding for benzene, and δ_{benz} is the experimental H NMR shift (7.3 ppm) of benzene.

6. Computed GIMIC ring current plots

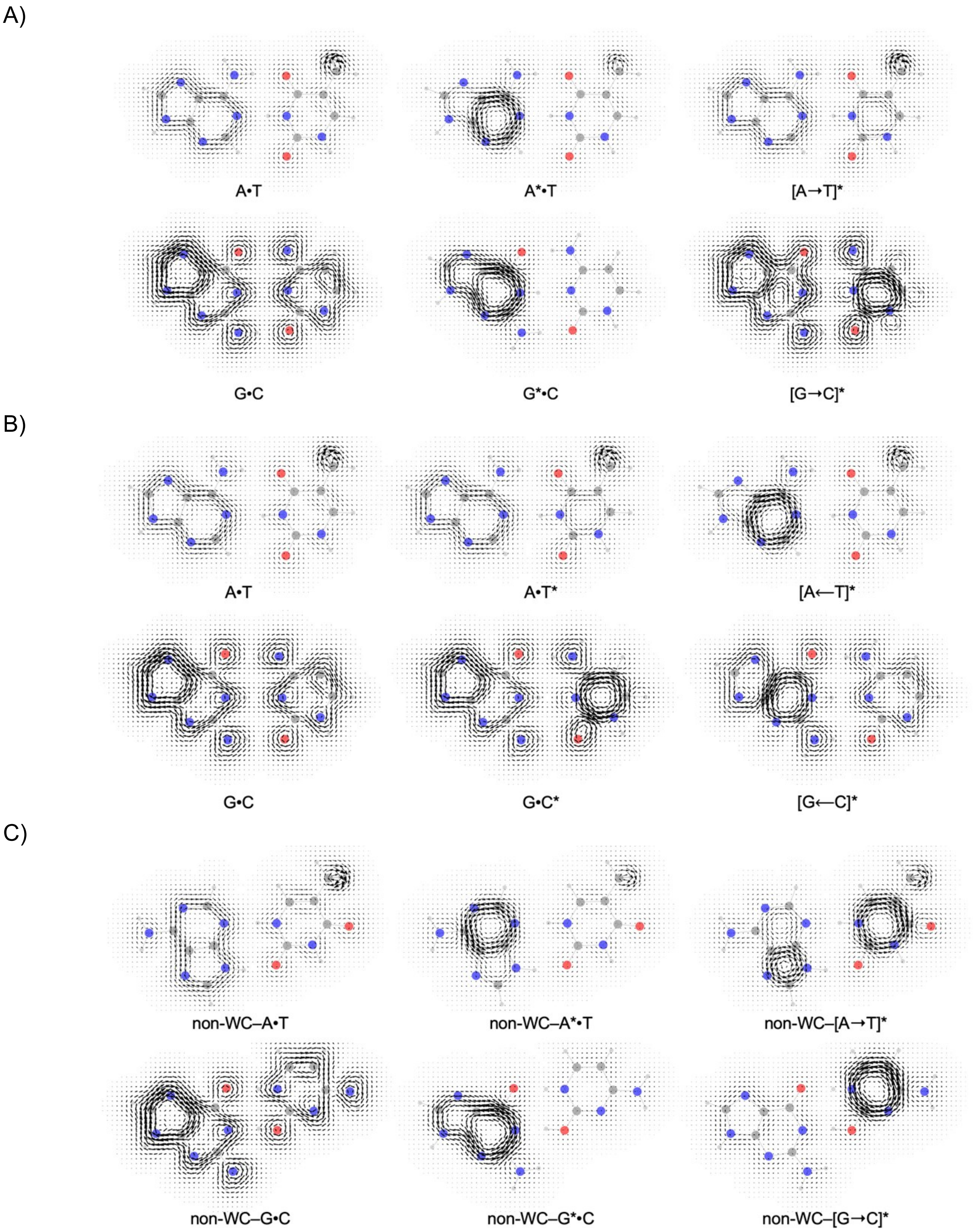

Figure S5. Computed GIMIC ring current plots for the GS (left column), LE (middle column), and CT (right column) states of the: A) Watson–Crick A•T and G•C pairs (excitation on purines), B) Watson–Crick A•T and G•C pairs (excitation on the pyrimidines), and C) non-Watson–Crick A•T and G•C pairs (excitation on purines).

7. Computed Harmonic Oscillator Model of Electron Delocalization (HOMED)

Table S7. HOMED values calculated at ωB97XD/6-311+G(d,p) for the Watson-Crick and non-Watson-Crick forms of A•T and G•C in different electronic states.

Base Pair Structure	purine	pyrimidine
A•T	0.923	0.740
A*•T	0.828	0.742
[A→T]*	0.918	0.772
G•C	0.845	0.834
G*•C	0.821	0.866
[G→C]*	0.841	0.773
A•T	0.923	0.740
A•T*	0.922	0.716
[A⟵T]*	0.750	0.753
G•C	0.845	0.834
G•C*	0.836	0.814
[G⟵C]*	0.817	0.817
non-WC A•T	0.940	0.732
non-WC A*•T	0.820	0.736
non-WC [A→T]*	0.904	0.511
non-WC G•C	0.854	0.876
non-WC G*•C	0.812	0.872
non-WC [G→C]*	0.847	0.769

HOMED values were computed to quantify the effects of bond length alternation in the GS, LE, and CT states of the isolated base pairs. When excitation happens on the purines (A or G), the purine fragments show increased bond length alternation in the LE states (lower HOMED values compared to the GS states), and following PCET, the CT states regain bond length equalization character (HOMED values closer to the GS states). Note changes in the pyrimidine fragments; similar values for the GS and LE states, and increased bond length equalization in the CT state. When excitation happens on the pyrimidines (T or C), the purine fragments show minimal bond length alternation in the LE states (HOMED values close to the GS states), but much increased bond length alternation in the CT states (lower HOMED values compared to the GS states). Note also changes in the pyrimidine fragments; increased bond length alternation in the LE state, and bond length equalization in the CT state.

8. Multicenter indices (MCI) values

Multicenter indices (MCI) values were calculated for the C_s geometries of base pairs, in the GS, LE, and CT states, at the CASSCF/6-311+G(d,p) level with an active space of eight π-orbitals (four occupied and four unoccupied π-orbitals, with eight electrons). MCI values were computed for the complete base pair structures (instead of base fragments), and show a loss of aromaticity upon excitation, with gain in aromaticity upon proton-coupled electron transfer. The MCI quantifies the extent of delocalized cyclic bonding—larger values reflect a more aromatic ring. For example, the MCI for benzene is 0.0435 at the CASSCF(6,6)/6-311++G(d,p) level.

Table S8. Computed MCI values at the CASSCF(8,8)/6-311+G(d,p) level for A•T.

Base pair structure	Adenine (6MR)	Adenine (5MR)	Thymine
WC A•T (excitation on purine)			
A•T	0.0196	0.0153	0.0018
A*•T	0.0066	0.0129	0.0018
[A→T]*	0.0130	0.0193	0.0033
WC A•T (excitation on pyrimidine)			
A•T	0.0196	0.0153	0.0018
A•T*	0.0177	0.0166	0.0021
[A←T]*	0.0073	0.0155	0.0019
non-WC A•T (excitation on purine)			
non-WC A•T	0.0185	0.0194	0.0021
non-WC A*•T	0.0064	0.0119	0.0022
non-WC [A→T]*	0.0088	0.0155	0.0054

Table S9. Computed MCI values at the CASSCF(8,8)/6-311+G(d,p) level for G•C.

Base pair structure	Adenine (6MR)	Adenine (5MR)	Thymine
WC G•C (excitation on purine)			
G•C	0.0054	0.0188	0.0058
G*•C	0.0029	0.0091	0.0069
[G→C]*	0.0062	0.0131	0.0040
WC G•C (excitation on pyrimidine)			
G•C	0.0054	0.0188	0.0058
G•C*	0.0060	0.0185	0.0046
[G←C]*	0.0055	0.0149	0.0050
non-WC G•C (excitation on purine)			
non-WC G•C	0.0057	0.0191	0.0090
non-WC G*•C	0.0031	0.0090	0.0088
non-WC [G→C]*	0.0063	0.0132	0.0096
WC G•C (alternative PCET)			
G•C	0.0054	0.0188	0.0058
Alternative G*•C	0.0036	0.0119	0.0067

Alternative [G→C]*	0.0032	0.0139	0.0074

9. Computed MCI values for Watson–Crick A•T and G•C structures at C_1 symmetry

Since minima geometries of the LE and CT states of WC A•T and WC G•C are nonplanar, we also considered the effects of aromaticity gain and loss based on the C_1 structures. Minima geometries, energies, and MCI values of the GS, LE, and CT states were computed at the (TD-)ωB97XD/6-311+G(d,p) level and the trends agree with that observed for the planar models. Upon excitation to the LE state (A*•T and G*•C), both base pairs show a loss of aromatic character in the six membered ring of the purine. Crossing to the CT state ([A→T]* and [G→C]*) regains a large part of the aromatic character (Figure S4). Note the near planar geometry of the purines in base pairs at the CT state, indicating re-aromatization (Figure S5).

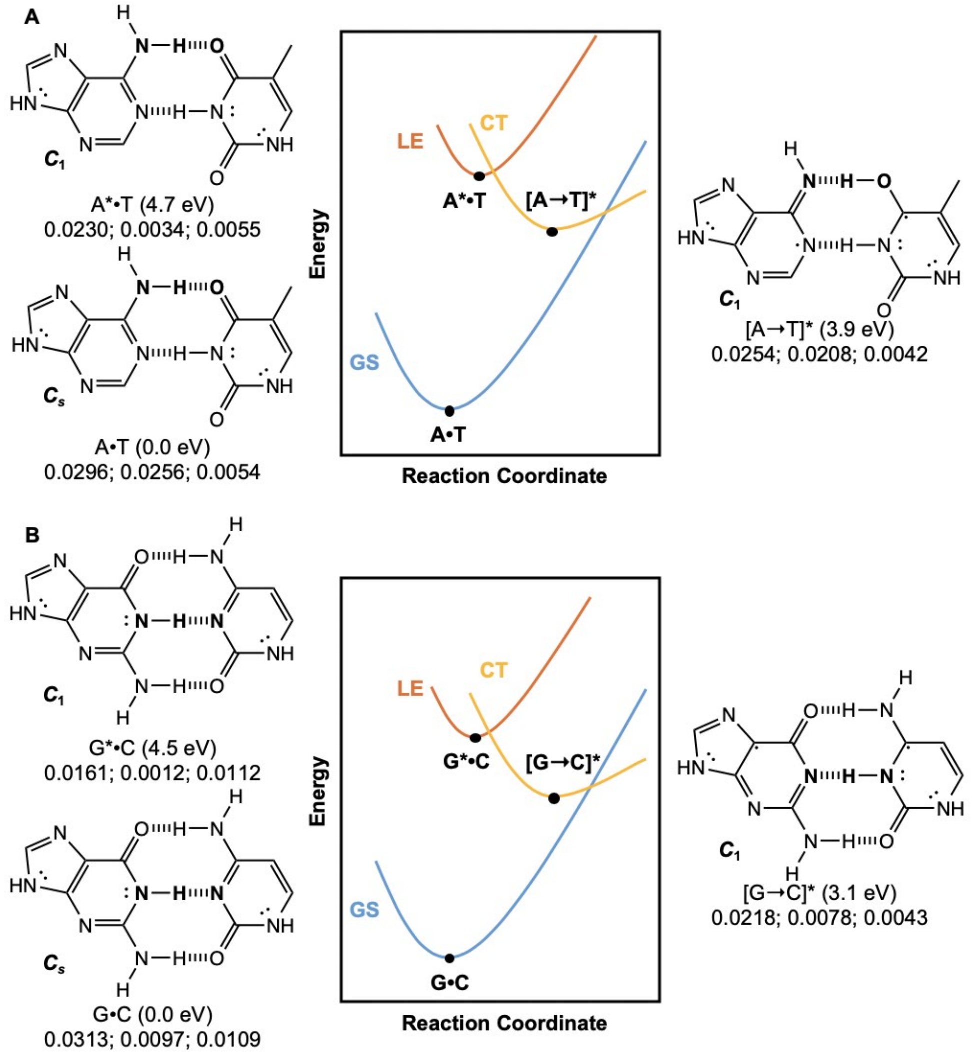

Figure S6. Energies and MCI values based on minima geometries of the GS, LE, and CT states, for: A) WC A•T, and B) WC G•C, at (TD-)ωB97XD/6-311+G(d,p).

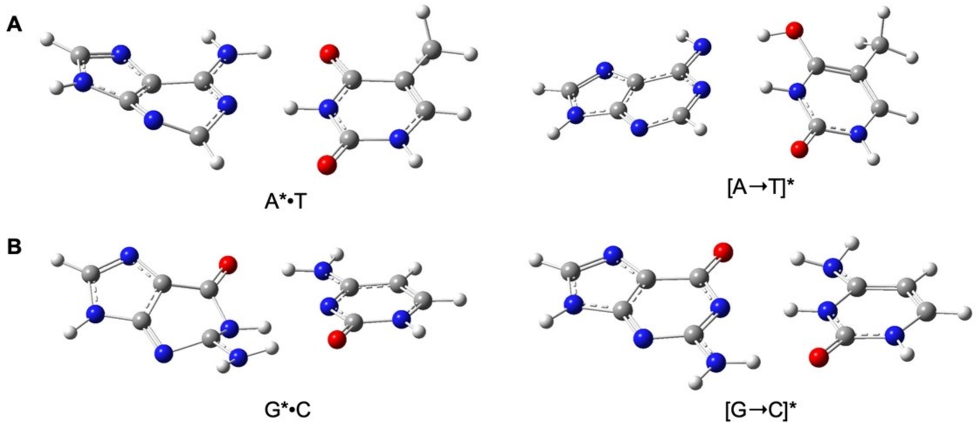

Figure S7. Minima geometries for the LE and CT states of: A) WC A•T and B) WC G•C.

10. Alternative proton-coupled electron transfer pathway in the Watson–Crick G•C base pair.

We also considered an alternative PCET pathway in the WC G•C pair, involving excitation on the purine, and proton transfer from the exocyclic NH_2 group. Compared to transfer of the pyridinium proton, the resulting CT structure is higher in energy (3.0 eV relative to ground state G•C, compared to 2.6 eV for the pyridinium proton transfer), and computed NICS(1)$_{zz}$ values suggest less antiaromaticity relief (Figure S6). Consistent with our findings, studies based on dynamics simulations reported an expected longer excited-state lifetime for this proton transfer route. See: Francés-Monerris, A., Gattuso, H., Roca-Sanjuán, D., Tuñón, I., Marazzi, M., Dumont, E. & Monari, A. Dynamics of the excited-state hydrogen transfer in a (dG)(dC) homopolymer: intrinsic photostability of DNA. *Chem. Sci.* **9**, 7902–7911 (2018).

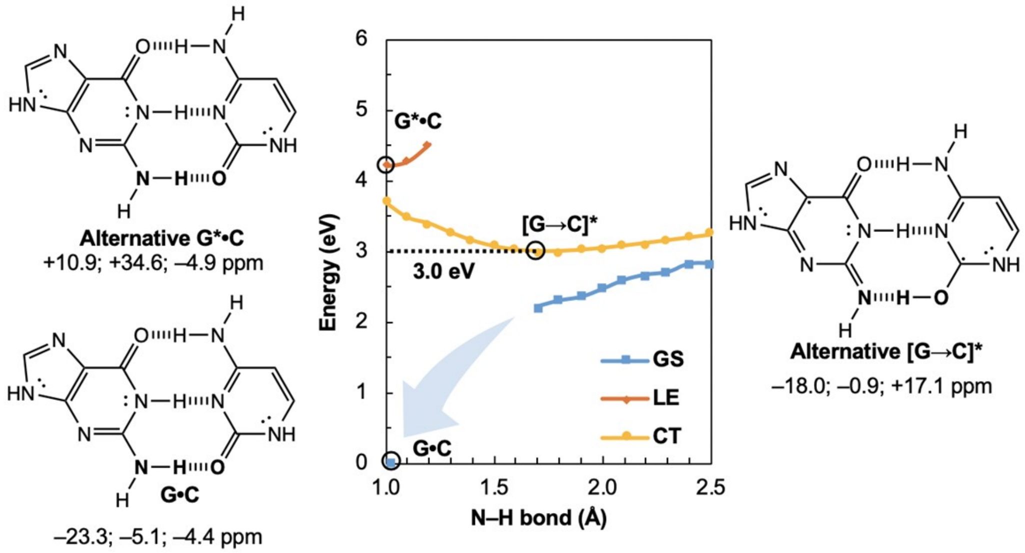

Figure S8. Potential energy functions of the electronic ground state (GS), locally-excited $^1\pi\pi^*$ state (LE), and charge-transfer state (CT), with constrained N–H bond distance, for the Watson-Crick structure of G•C involving the exocyclic NH_2 of guanine at CASPT2(8,8)/6-311+G(d,p). NICS(1)$_{zz}$ values were computed for relevant structures at the CASSCF(10,10)/6-311+G(d,p) level.

11. Homolytic N–H σ-bond cleavage of adenine and guanine.

Computed energy profile for the homolytic N–H σ-bond cleavage of adenine and guanine at CASPT2(10,10)/6-311+G(d,p), in the ground state (GS) and $^1\pi\pi^*$ excited state (LE). Note that, for both adenine and guanine, the GS and LE curves for σ-bond cleavage parallel each other and are highly endothermic.

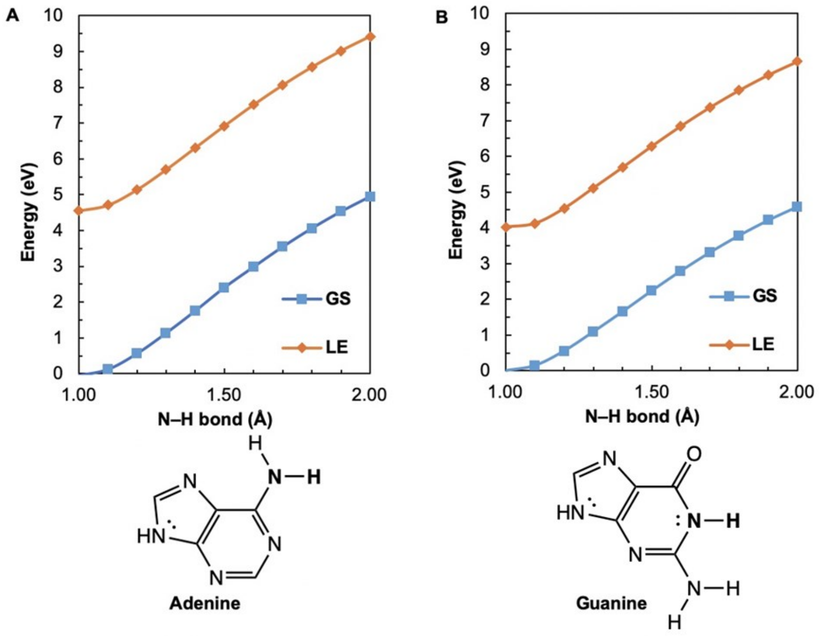

Figure S9. Potential energy functions for constrained N–H stretching (see N–H bond in bold) of the ground state (GS) and locally-excited $^1\pi\pi^*$ state (LE) of A) adenine and B) guanine at CASPT2(10,10)/6-311+G(d,p).

12. Computed vertical ionization potentials (IP) and electron affinities (EA)

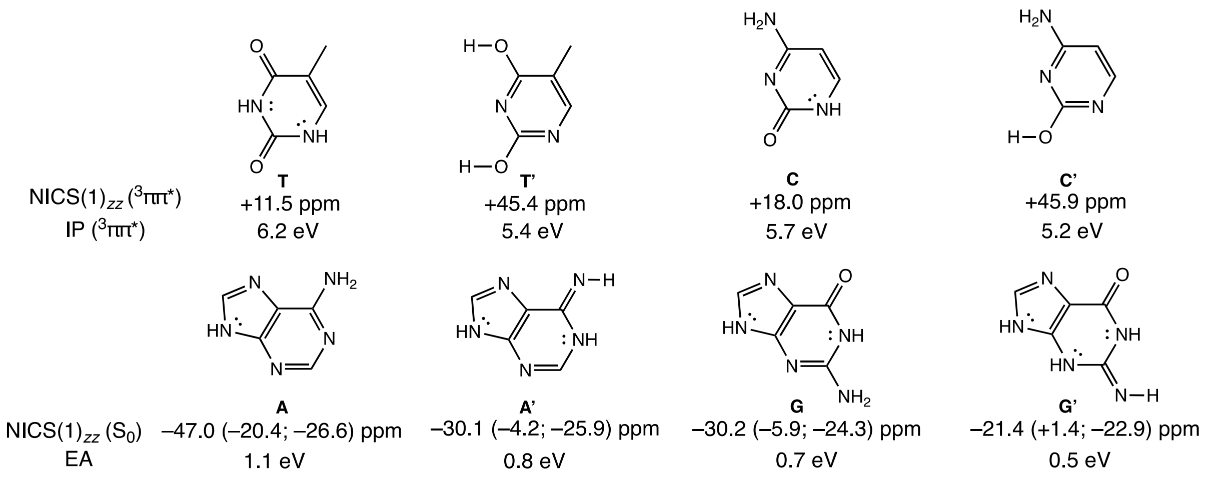

Figure S10. Computed vertical ionization potentials (IP) in the $^{3}\pi\pi^{*}$ states of pyrimidines (**T** and **C**) and vertical electron affinities (EA) in the ground states of purines (**A** and **G**), compared to their isomers (**A’**, **G’**, **T’**, **C’**) at ωB97X-D/6-311+G(d,p). Dissected NICS(1)$_{zz}$ values were computed at PW91/IGLOIII.

We show in Figure S10 that the power to photo-oxidize a nucleobase (as evaluated by the triplet state IPs) and to reduce a nucleobase (as evaluated by the ground state EAs), can be related to, respectively, the excited-state antiaromatic character and the ground state aromatic character of the nucleobase considered. As shown in Figure S10 (top row), the higher triplet state IPs of thymine (**T**) ($^{3}\pi\pi^{*}$ state IP: 6.2 eV) and cytosine (**C**) ($^{3}\pi\pi^{*}$ state IP: 5.7 eV), compared to their fully cyclic [4n+2] π-electron delocalized isomers **T’** ($^{3}\pi\pi^{*}$ state IP: 5.4 eV) and **C’** ($^{3}\pi\pi^{*}$ state IP: 5.2 eV), can be rationalized by weaker excited-state antiaromaticity in the $^{3}\pi\pi^{*}$ states of **T** and **C** (note more positive NICS(1)$_{zz}$ values in **T’** and **C’** than in **T** and **C**). Also (bottom row), the higher ground state EAs of thymine (**A**) (EA: 1.1 eV) and guanine (**G**) (EA: 0.7 eV) (i.e., difficulty to add an electron to the base), compared to their fully cyclic [4n+2] π-electron delocalized isomers **A’** (EA: 0.8 eV) and **G’** (EA: 0.5 eV), can be rationalized by greater aromaticity in the ground states of **A** and **G** (note more negative NICS(1)$_{zz}$ values for of **A** and **G**).

13. DNA duplex model study

Excited-state proton-coupled electron transfer in a G•C:C•G DNA duplex was considered, following the deactivation pathway proposed by Kohler and coworkers in *J. Am. Chem. Soc.* **137**, 7059–7062 (2015) (see Figure S7). The studied G•C:C•G duplex structure was modified from a PDB file (1BNA) and computed using an ONIOM scheme. All four nucleobase structures were optimized at ωB97XD/6-31+G(d). The sugar phosphate backbone (see Figure S11, in grey) was optimized using a semiempirical method, PM6, with fixed atomic positions for all heavy atoms. Relative energies and multicenter index (MCI) analyses were computed at ωB97XD/6-31+G(d) for the resulting ONIOM optimized geometries.

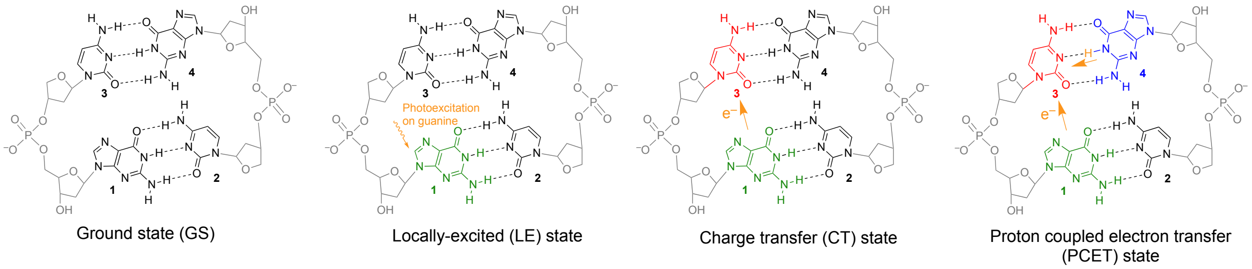

Figure S11. Proton-coupled electron transfer in the G•C:C•G DNA duplex.

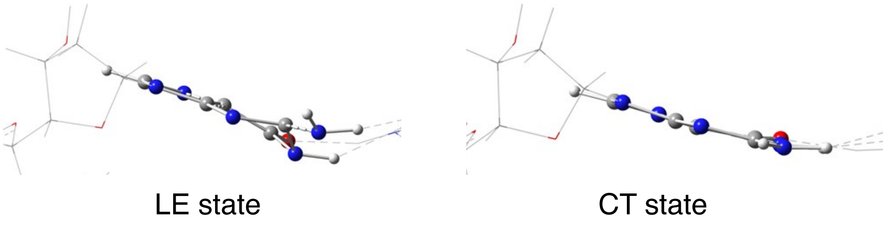

Figure S12. Optimized structure of guanine **1** in the LE state and CT state. Note that **1** is non-planar in the LE state, but becomes planar upon electron transfer to cytosine **3**.

Upon local excitation on guanine **1** (LE state), the energy of the CT and PCET states lower as an electron is transferred from guanine **1** to cytosine **3** through base stacking, and as a proton is transferred from guanine **4** to cytosine **3** (see relative energies in Table S10). In the LE state, guanine **1** (green color in LE state) loses aromaticity (note lower MCI value, Table S11) and planarity (Figure S12). Transferring an electron to cytosine **3** (red color in CT state) regains some aromatic character restores planarity (see Table S11 and Figure S11). Cytosine **3** loses a lesser degree of aromaticity) upon accepting an electron (cf. MCI value for **1** in the LE state.

Table S10. Relative energies (in eV) for the ground state, locally-excited (LE) state, charge transfer (CT) state, and proton-coupled electron transfer state (PCET) of the G•C:C•G duplex at (TD-)ωB97XD/6-31+G(d)//ONIOM(ωB97XD/6-31+G(d):PM6).

State	Relative energy (eV)
Ground state	0
LE state	4.65
CT state	4.18
PCET state	3.93

Table S11. Computed multicenter index (MCI) values for each ring system, at (TD-)ωB97XD/6-31+G(d)//ONIOM (ωB97XD/6-31+G(d):PM6).

	$\mathbf{1}_{6MR}$	$\mathbf{1}_{5MR}$	**2**	**3**	$\mathbf{4}_{6MR}$	$\mathbf{4}_{5MR}$
GS	0.0098	0.0287	0.0117	0.0120	0.0094	0.0288
LE state	0.0015	0.0153	0.0122	0.0116	0.0096	0.0288
CT state	0.0052	0.0188	0.0123	0.0062	0.0093	0.0287
PCET state	0.0054	0.0193	0.0125	0.0044	0.0144	0.0277

14. References for Software and Programs

Gaussian 16 Revision A.03:
Frisch, M. J., Trucks, G. W., Schlegel, H. B., Scuseria, G. E., Robb, M. A., Cheeseman, J. R., Scalmani, G., Barone, V., Petersson, G. A., Nakatsuji, H., Li, X., Caricato, M., Marenich, A. V., Bloino, J., Janesko, B. G., Gomperts, R., Mennucci, B., Hratchian, H. P., Ortiz, J. V., Izmaylov, A. F., Sonnenberg, J. L., Williams-Young, D., Ding, F., Lipparini, F., Egidi, F., Goings, J., Peng, B., Petrone, A., Henderson, T., Ranasinghe, D., Zakrzewski, V. G., Gao, J., Rega, N., Zheng, G., Liang, W., Hada, M., Ehara, M., Toyota, K., Fukuda, R., Hasegawa, J., Ishida, M., Nakajima, T., Honda, Y., Kitao, O., Nakai, H., Vreven, T., Throssell, K., Montgomery, J. A., Jr., Peralta, J. E., Ogliaro, F., Bearpark, M. J., Heyd, J. J., Brothers, E. N., Kudin, K. N., Staroverov, V. N., Keith, T. A., Kobayashi, R., Normand, J., Raghavachari, K., Rendell, A. P., Burant, J. C., Iyengar, S. S., Tomasi, J., Cossi, M., Millam, J. M., Klene, M., Adamo, C., Cammi, R., Ochterski, J. W., Martin, R. L., Morokuma, K., Farkas, O., Foresman, J. B., Fox, D. J. *Gaussian 16, Revision A.03* (Gaussian, Inc., Wallingford CT, 2016).

Multiwfn:
Lu, T. & Chen, F. Multiwfn: A multifunctional wavefunction analyzer. *J. Comput. Chem.* **33**, 580-592 (2012).

Molpro 2012.1:
Werner, H.-J., Knowles, P. J., Knizia, G. Manby, F. R. & Schütz M. Molpro: a general-purpose quantum chemistry program. *WIREs Comput. Mol. Sci.* **2**, 242-253 (2012).

Dalton 2016.2:
K. Aidas, C. Angeli, K. L. Bak, V. Bakken, R. Bast, L. Boman, O. Christiansen, R. Cimiraglia, S. Coriani, P. Dahle, E. K. Dalskov, U. Ekström, T. Enevoldsen, J. J. Eriksen, P. Ettenhuber, B. Fernández, L. Ferrighi, H. Fliegl, L. Frediani, K. Hald, A. Halkier, C. Hättig, H. Heiberg, T. Helgaker, A. C. Hennum, H. Hettema, E. Hjertenæs, S. Høst, I.-M. Høyvik, M. F. Iozzi, B. Jansik, H. J. Aa. Jensen, D. Jonsson, P. Jørgensen, J. Kauczor, S. Kirpekar, T. Kjærgaard, W. Klopper, S. Knecht, R. Kobayashi, H. Koch, J. Kongsted, A. Krapp, K. Kristensen, A. Ligabue, O. B. Lutnæs, J. I. Melo, K. V. Mikkelsen, R. H. Myhre, C. Neiss, C. B. Nielsen, P. Norman, J. Olsen, J. M. H. Olsen, A. Osted, M. J. Packer, F. Pawlowski, T. B. Pedersen, P. F. Provasi, S. Reine, Z. Rinkevicius, T. A. Ruden, K. Ruud, V. Rybkin, P. Salek, C. C. M. Samson, A. Sánchez de Merás, T. Saue, S. P. A. Sauer, B. Schimmelpfennig, K. Sneskov, A. H. Steindal, K. O. Sylvester-Hvid, P. R. Taylor, A. M. Teale, E. I. Tellgren, D. P. Tew, A. J. Thorvaldsen, L. Thøgersen, O. Vahtras, M. A. Watson, D. J. D. Wilson, M. Ziolkowski, and H. Ågren, "The Dalton quantum chemistry program system", *WIREs Comput. Mol. Sci.* **4**, 269–284 (2014).

Dalton, a molecular electronic structure program, Release v2016.2 (2017), see http://daltonprogram.org.

ESI-3D
Matito, E. ESI-3D: Electron Sharing Indices Program for 3D Molecular Space Partitioning; Institute of Computational chemistry and Catalysis (IQCC), University of Girona, Catalonia, Spain, 2006; http://iqc.udg.es/ eduard/ESI

15. CASPT2(8,8)/6-311+G(d,p) energies for point on the potential energy curves.

Table S12. Computed total electronic energies (in Hartree) for the GS, LE, and CT states of WC A•T, as a function of N–H bond stretching (see bold-type N–H bond in Figure 2A).

N–H bond (Å)	GS	LE	CT
Equilibrium	-919.2237	—	—
1.0	—	-919.0506	-919.0526
1.1	—	-919.0397	-919.0662
1.2	—	-919.0278	-919.0768
1.3	—	-919.0496	-919.0860
1.4	—	—	-919.0934
1.5	—	—	-919.0988
1.6	—	—	-919.1025
1.7	—	—	-919.1049
1.8	—	—	-919.1062
1.9	-919.1415	—	-919.1068
2.0	-919.1377	—	-919.1045
2.1	-919.1333	—	-919.1044
2.2	-919.1293	—	-919.1041
2.3	-919.1219	—	-919.1037
2.4	-919.1179	—	-919.1032
2.5	-919.1137	—	-919.1027

Table S13. Computed total electronic energies (in Hartree) for the GS, LE, and CT states of WC A•T (excitation on pyrimidine), as a function of N–H bond stretching (see bold-type N–H bond in Figure 3A).

N–H bond (Å)	GS	LE	CT
Equilibrium	-919.2237	—	—
1.0	—	-919.0593	—
1.1	—	-919.0594	—
1.2	—	-919.0541	—
1.3	—	-919.0500	—
1.4	—	—	—
1.5	—	—	—
1.6	—	—	—
1.7	—	—	-919.0264
1.8	—	—	-919.0298
1.9	—	—	-919.0319
2.0	—	—	-919.0330
2.1	-919.1649	—	-919.0335
2.2	-919.1601	—	-919.0336
2.3	-919.1544	—	-919.0317
2.4	-919.1520	—	-919.0309
2.5	-919.1494	—	-919.0303

Table S14. Computed total electronic energies (in Hartree) for the GS, LE, and CT states of non-WC A•T, as a function of N–H bond stretching (see bold-type N–H bond in Figure 4A).

N–H bond (Å)	GS	LE	CT
Equilibrium	-919.2301	—	—
1.0	—	-919.0547	—
1.1	—	-919.0530	—
1.2	—	-919.0365	-919.0390
1.3	—	—	-919.0457
1.4	—	—	-919.0512
1.5	—	—	-919.0551
1.6	—	—	-919.0575
1.7	—	—	-919.0577
1.8	-919.1627	—	-919.0591
1.9	-919.1594	—	-919.0589
2.0	-919.1560	—	-919.0571
2.1	-919.1494	—	-919.0574
2.2	-919.1462	—	-919.0565
2.3	-919.1433	—	-919.0555
2.4	-919.1404	—	-919.0545
2.5	-919.1376	—	-919.0535

Table S15. Computed total electronic energies (in Hartree) for the GS, LE, and CT states of WC G•C, as a function of N–H bond stretching (see bold-type N–H bond in Figure 2B).

N–H bond (Å)	GS	LE	CT
Equilibrium	-935.2596	—	—
1.0	—	-935.1007	-935.1229
1.1	—	-935.1002	-935.1281
1.2	—	—	-935.1322
1.3	—	—	-935.1383
1.4	—	—	-935.1455
1.5	—	—	-935.1520
1.6	—	—	-935.1570
1.7	—	—	-935.1605
1.8	—	—	-935.1626
1.9	-935.1869	—	-935.1634
2.0	-935.1827	—	-935.1631
2.1	-935.1780	—	-935.1621
2.2	-935.1706	—	-935.1606
2.3	-935.1652	—	-935.1587
2.4	-935.1571	—	-935.1567
2.5	-935.1481	—	-935.1546

Table S16. Computed total electronic energies (in Hartree) for the GS, LE, and CT states of WC G•C (excitation on pyrimidine), as a function of N–H bond stretching (see bold-type N–H bond in Figure 3B).

N–H bond (Å)	GS	LE	CT
Equilibrium	-935.2596	—	—
1.0	—	-935.1088	—
1.1	—	-935.1054	—
1.2	—	-935.0987	—
1.3	—	-935.0875	—
1.4	—	—	—
1.5	—	—	—
1.6	—	—	-935.0734
1.7	—	—	-935.0783
1.8	—	—	-935.0779
1.9	—	—	-935.0806
2.0	—	—	-935.0808
2.1	-935.1929	—	-935.0789
2.2	-935.1892	—	-935.0784
2.3	-935.1857	—	-935.0778
2.4	-935.1820	—	-935.0770
2.5	-935.1784	—	-935.0759

Table S17. Computed total electronic energies (in Hartree) for the GS, LE, and CT states of non-WC G•C, as a function of N–H bond stretching (see bold-type N–H bond in Figure 4B).

N–H bond (Å)	GS	LE	CT
Equilibrium	-935.2543	—	—
1.0	—	-935.0986	—
1.1	—	-935.0976	—
1.2	—	-935.0909	-935.1071
1.3	—	—	-935.1129
1.4	—	—	-935.1180
1.5	—	—	-935.1217
1.6	—	—	-935.1238
1.7	—	—	-935.1248
1.8	-935.1842	—	-935.1247
1.9	-935.1823	—	-935.1241
2.0	-935.1803	—	-935.1231
2.1	-935.1782	—	-935.1219
2.2	-935.1762	—	-935.1207
2.3	-935.1739	—	-935.1195
2.4	-935.1718	—	-935.1183
2.5	-935.1695	—	-935.1171

Table S18. Computed total electronic energies (in Hartree) for the GS, LE, and CT states of WC G•C (alternative PCET pathway), as a function of N–H bond stretching (see bold-type N–H bond in Figure S6).

N–H bond (Å)	GS	LE	CT
Equilibrium	-935.2596	—	—
1.0	—	-935.1048	-935.1237
1.1	—	-935.1026	-935.1316
1.2	—	-935.0941	-935.1347
1.3	—	—	-935.1395
1.4	—	—	-935.1437
1.5	—	—	-935.1468
1.6	—	—	-935.1486
1.7	-935.1782	—	-935.1494
1.8	-935.1752	—	-935.1493
1.9	-935.1733	—	-935.1487
2.0	-935.1690	—	-935.1477
2.1	-935.1643	—	-935.1465
2.2	-935.1615	—	-935.1451
2.3	-935.1602	—	-935.1436
2.4	-935.1558	—	-935.1422
2.5	-935.1556	—	-935.1408

16. Optimized Cartesian coordinates for relevant structures.

WC A•T (C_s)

	X	Y	Z
C	-2.11184	-0.73537	0.00000
C	1.83469	1.88203	0.00000
N	-0.76941	-0.70388	0.00000
N	0.45369	1.84824	0.00000
C	-0.07423	-1.85456	0.00000
C	-0.39969	2.92550	0.00000
C	-0.81199	-3.05494	0.00000
N	0.25257	4.14640	0.00000
N	-2.90148	-1.79646	0.00000
C	2.44465	3.21174	0.00000
C	-2.19362	-2.93129	0.00000
C	1.62023	4.27387	0.00000
N	-2.65591	-4.22141	0.00000
N	-0.42553	-4.37997	0.00000
C	-1.54972	-5.03681	0.00000
N	1.26268	-1.81980	0.00000
H	-1.64181	-6.11309	0.00000
H	-2.58953	0.24045	0.00000
O	2.48366	0.84338	0.00000
H	1.77148	-2.68649	0.00000
H	1.75693	-0.92822	0.00000
H	0.00001	0.90644	0.00000
O	-1.60763	2.83172	0.00000
H	1.99003	5.29249	0.00000
H	-3.62257	-4.50110	0.00000
H	-0.34502	4.95675	0.00000
C	3.93798	3.31008	0.00000
H	4.35786	2.81284	0.87778
H	4.35786	2.81284	-0.87778
H	4.26161	4.35292	0.00000

N_{Im}= 0

WC A*•T (C_s)

	X	Y	Z
N	-0.06190	-2.32303	0.00000
C	-0.77035	-3.45701	0.00000
N	-2.17623	-3.55764	0.00000
C	-2.75917	-2.40214	0.00000
N	-4.11342	-2.09158	0.00000
C	-4.21717	-0.71351	0.00000
N	-3.00993	-0.11339	0.00000
C	-2.13367	-1.11643	0.00000
C	-0.68620	-1.14717	0.00000
N	-0.00742	0.00752	0.00000
N	2.79428	-2.49744	0.00000
C	3.45629	-1.28603	0.00000
C	4.91589	-1.34887	0.00000
C	5.48521	-2.56765	0.00000
N	4.74832	-3.72544	0.00000
C	3.36346	-3.75126	0.00000
O	2.82958	-0.23143	0.00000
C	5.68526	-0.06507	0.00000
O	2.73022	-4.78165	0.00000
H	-0.21784	-4.38628	0.00000
H	-4.86664	-2.75759	0.00000
H	-5.16456	-0.19940	0.00000
H	-0.53446	0.86772	0.00000
H	0.99256	0.01347	0.00000
H	1.75343	-2.46845	0.00000
H	6.56040	-2.70281	0.00000
H	5.19695	-4.62701	0.00000
H	5.43538	0.53552	0.87798
H	6.76065	-0.25386	0.00000
H	5.43538	0.53552	-0.87798

N_{Im}= 2 (424*i* and 44*i* cm^{-1})

WC [A →T]* (C_s)

	X	Y	Z
C	-0.63276	-3.40556	0.00000
N	0.03019	-2.27092	0.00000
C	-0.63545	-1.07473	0.00000
C	-2.05635	-1.13800	0.00000
N	-1.97678	-3.57056	0.00000
C	-2.62290	-2.42503	0.00000
N	-3.97958	-2.20115	0.00000
N	-3.02117	-0.17468	0.00000
C	-4.14694	-0.84696	0.00000
N	0.09834	0.01953	0.00000
H	-5.12903	-0.39784	0.00000
H	-0.03278	-4.30860	0.00000
H	-0.51369	0.83648	0.00000
H	-4.69806	-2.90656	0.00000
C	3.71024	-1.05197	0.00000
N	2.95812	-2.23020	0.00000
C	3.50725	-3.49093	0.00000
N	4.87335	-3.51216	0.00000
C	5.08994	-1.11612	0.00000
C	5.68273	-2.36063	0.00000
O	3.05476	0.12498	0.00000
H	1.94614	-2.21007	0.00000
O	2.82224	-4.50084	0.00000
H	6.74776	-2.52165	0.00000
H	5.28389	-4.42883	0.00000
C	5.90639	0.14467	0.00000
H	5.68841	0.75430	-0.87988
H	5.68841	0.75430	0.87988
H	6.97213	-0.09058	0.00000
H	2.09834	0.01782	0.00000

N_{Im}= 1 (26*i* cm^{-1})

WC A•T* (C_s)

	X	Y	Z
C	3.51151	-1.13195	0.00000
N	2.84270	0.03031	0.00000
C	3.52701	1.18979	0.00000
C	4.93528	1.10951	0.00000
N	4.82081	-1.32622	0.00000
C	5.48359	-0.16414	0.00000
N	6.83873	0.04212	0.00000
N	5.91745	2.07931	0.00000
C	7.03022	1.40298	0.00000
N	2.86467	2.34931	0.00000
H	8.02081	1.83376	0.00000
H	2.88541	-2.01991	0.00000
H	3.39143	3.20546	0.00000
H	1.83967	2.36870	0.00000
H	7.54427	-0.67528	0.00000
N	-2.04999	-1.17184	0.00000
C	-2.81554	-0.04082	0.00000
H	-3.88951	-0.14132	0.00000
C	-2.10710	1.20921	0.00000
C	-2.85280	2.48320	0.00000
C	-0.67977	1.24214	0.00000
O	0.00593	2.28422	0.00000
N	-0.01856	-0.01263	0.00000
H	0.98142	-0.01887	0.00000
C	-0.65075	-1.20972	0.00000
O	-0.09382	-2.29387	0.00000
H	-2.47289	-2.09021	0.00000
H	-2.57116	3.08877	0.87074
H	-2.57116	3.08877	-0.87074
H	-3.93271	2.32709	0.00000

N_{Im}= 4 (301*i*, 187*i*, 118*i*, and 66*i* cm^{-1})

WC [A→T]* (C_s)

	X	Y	Z
C	3.60287	-1.45809	0.00000
N	3.08697	-0.12368	0.00000
C	3.87895	0.97162	0.00000
C	5.23758	0.73859	0.00000
N	4.94804	-1.69516	0.00000
C	5.67196	-0.62086	0.00000
N	7.05637	-0.50213	0.00000
N	6.31486	1.61995	0.00000
C	7.35373	0.85019	0.00000
N	3.32158	2.19957	0.00000
H	8.37772	1.19524	0.00000
H	2.88700	-2.25349	0.00000
H	3.93708	2.99262	0.00000
H	2.32417	2.33405	0.00000
H	7.70761	-1.26624	0.00000
N	-2.16081	-1.00127	0.00000
C	-2.76250	0.22199	0.00000
H	-3.84546	0.21640	0.00000
C	-2.04470	1.36903	0.00000
C	-2.65924	2.72932	0.00000
C	-0.58833	1.26553	0.00000
O	0.15253	2.23431	0.00000
N	-0.00780	-0.01178	0.00000
C	-0.78326	-1.11838	0.00000
O	-0.26756	-2.24202	0.00000
H	-2.69496	-1.85563	0.00000
H	2.09219	-0.01401	0.00000
H	-3.74900	2.67726	0.00000
H	-2.32967	3.29340	0.87610
H	-2.32967	3.29340	-0.87610

N_{Im}= 2 (425*i* and 75*i* cm^{-1})

non-WC A•T (C_s)

	X	Y	Z
N	1.74918	1.31393	0.00000
C	0.58241	2.03122	0.00000
H	0.58871	3.11124	0.00000
N	-0.49360	1.29033	0.00000
C	-0.00361	0.00225	0.00000
C	-0.62922	-1.25565	0.00000
N	-1.96727	-1.39044	0.00000
H	-2.36795	-2.31184	0.00000
H	-2.55563	-0.57587	0.00000
N	0.13426	-2.35758	0.00000
C	1.45897	-2.21475	0.00000
H	2.02795	-3.13988	0.00000
N	2.16829	-1.08997	0.00000
C	1.38444	0.00075	0.00000
H	2.71890	1.64908	0.00000
N	4.99463	-0.50955	0.00000
C	5.93306	-1.51310	0.00000
H	5.51889	-2.51465	0.00000
C	7.25989	-1.29086	0.00000
C	8.29272	-2.37385	0.00000
C	7.71927	0.09711	0.00000
O	8.87679	0.45564	0.00000
N	6.68723	1.04610	0.00000
H	6.96861	2.01688	0.00000
C	5.33445	0.81096	0.00000
O	4.51998	1.72669	0.00000
H	3.98693	-0.74935	0.00000
H	7.82491	-3.36022	0.00000
H	8.93837	-2.29160	-0.87777
H	8.93837	-2.29160	0.87777

N_{Im}= 0

non-WC A*•T (C_s)

	X	Y	Z
N	-0.00692	0.01049	0.00000
C	-0.87506	1.06027	0.00000
H	-0.54924	2.08789	0.00000
N	-2.16867	0.66138	0.00000
C	-2.10874	-0.66846	0.00000
C	-3.13116	-1.70439	0.00000
N	-4.42907	-1.33811	0.00000
H	-5.14907	-2.04244	0.00000
H	-4.67059	-0.35969	0.00000
N	-2.77009	-2.97868	0.00000
C	-1.48053	-3.29548	0.00000
H	-1.21864	-4.34441	0.00000
N	-0.37833	-2.38980	0.00000
C	-0.76639	-1.14261	0.00000
N	2.45236	-2.78004	0.00000
C	3.01321	-4.03327	0.00000
H	2.29539	-4.84542	0.00000
C	4.34082	-4.25503	0.00000
C	4.96452	-5.61546	0.00000
H	4.20064	-6.39543	0.00000
C	5.22643	-3.09264	0.00000
O	6.43811	-3.12948	0.00000
N	4.55918	-1.85953	0.00000
H	5.14097	-1.03312	0.00000
C	3.20310	-1.64263	0.00000
O	2.73040	-0.51125	0.00000
H	1.41834	-2.67752	0.00000
H	0.99304	0.01982	0.00000
H	5.60175	-5.74841	-0.87779
H	5.60175	-5.74841	0.87779

N_{Im}= 2 (273*i* and 34*i* cm^{-1})

non-WC [A→T]* (C_s)

	X	Y	Z
C	-1.43127	-3.27196	0.00000
C	5.50941	-2.40423	0.00000
N	-2.73215	-3.02646	0.00000
N	4.69551	-1.16348	0.00000
C	-3.13558	-1.74598	0.00000
C	3.36474	-1.14701	0.00000
C	-2.14849	-0.71064	0.00000
N	2.70079	-2.28052	0.00000
N	-0.39725	-2.39094	0.00000
C	4.75448	-3.57647	0.00000
C	-0.78019	-1.13861	0.00000
C	3.34390	-3.53431	0.00000
N	0.00290	-0.00671	0.00000
N	-2.22308	0.60140	0.00000
C	-0.87832	0.96599	0.00000
N	-4.43168	-1.47123	0.00000
H	-0.59774	2.00928	0.00000
H	-1.14977	-4.31790	0.00000
O	6.72678	-2.22487	0.00000
H	-4.74716	-0.51397	0.00000
H	5.20147	-0.29356	0.00000
O	2.78179	0.03935	0.00000
H	2.69613	-4.39075	0.00000
H	1.69305	-2.27201	0.00000
C	5.47844	-4.89020	0.00000
H	1.80290	-0.00698	0.00000
H	-5.09669	-2.22862	0.00000
H	4.77087	-5.72302	0.00000
H	6.12274	-4.99076	-0.87846
H	6.12274	-4.99076	0.87846

N_{Im}= 4 (225*i*, 71*i*, 35*i*, and 10*i* cm^{-1})

WC G•C (C_s)

	X	Y	Z
N	4.31228	0.91516	0.00000
C	4.92093	-0.29747	0.00000
H	6.00363	-0.29639	0.00000
C	4.18619	-1.42735	0.00000
H	4.65195	-2.40205	0.00000
C	2.75261	-1.27382	0.00000
N	1.96235	-2.34374	0.00000
H	0.93459	-2.23473	0.00000
H	2.36050	-3.26580	0.00000
N	2.17679	-0.07390	0.00000
C	2.91938	1.05824	0.00000
O	2.44725	2.18910	0.00000
H	4.84264	1.77214	0.00000
N	-4.65042	0.46963	0.00000
C	-4.94357	-0.87953	0.00000
H	-5.96318	-1.23571	0.00000
N	-3.88272	-1.62672	0.00000
C	-2.83048	-0.73320	0.00000
C	-1.41925	-0.94459	0.00000
O	-0.79900	-2.00591	0.00000
N	-0.72260	0.26954	0.00000
H	0.30559	0.18608	0.00000
C	-1.28665	1.51759	0.00000
N	-0.43737	2.56080	0.00000
H	0.57627	2.44995	0.00000
H	-0.84500	3.47811	0.00000
N	-2.58676	1.72478	0.00000
C	-3.28992	0.57696	0.00000
H	-5.29725	1.24011	0.00000

N_{Im}= 0

WC G*•C (C_s)

	X	Y	Z
N	-4.01388	-0.07995	0.00000
C	-4.17598	-1.42810	0.00000
H	-5.15594	-1.88256	0.00000
N	-3.03905	-2.10906	0.00000
C	-2.06322	-1.16705	0.00000
C	-0.63898	-1.28498	0.00000
O	0.05831	-2.30304	0.00000
N	-0.00084	-0.00283	0.00000
H	0.99916	-0.00298	0.00000
C	-0.67208	1.18789	0.00000
N	0.05418	2.30209	0.00000
H	1.07416	2.29505	0.00000
H	-0.44984	3.17292	0.00000
N	-2.01702	1.30936	0.00000
C	-2.65236	0.15287	0.00000
H	-4.73677	0.61979	0.00000
N	4.83915	1.29964	0.00000
C	5.63688	0.18388	0.00000
H	6.70549	0.34666	0.00000
C	5.06048	-1.04570	0.00000
H	5.66524	-1.94173	0.00000
C	3.63865	-1.11010	0.00000
N	2.99245	-2.29205	0.00000
H	1.97198	-2.32411	0.00000
H	3.50839	-3.15219	0.00000
N	2.87896	0.00266	0.00000
C	3.44651	1.21786	0.00000
O	2.80445	2.27756	0.00000
H	5.22750	2.22801	0.00000

N_{Im}= 4 (406*i*, 162*i*, 68*i*, and 5*i* cm^{-1})

WC [G→C]* (C_s)

	X	Y	Z
N	-3.99262	0.23534	0.00000

C	-4.09120	1.59226	0.00000
H	-5.04717	2.09388	0.00000
N	-2.92402	2.21085	0.00000
C	-2.01175	1.22048	0.00000
C	-0.54810	1.23692	0.00000
O	0.09946	2.27393	0.00000
N	0.04101	-0.00224	0.00000
H	1.94099	-0.01001	0.00000
C	-0.68068	-1.11046	0.00000
N	-0.06184	-2.28826	0.00000
H	0.95663	-2.34422	0.00000
H	-0.62769	-3.11866	0.00000
N	-2.07183	-1.21798	0.00000
C	-2.64321	-0.05393	0.00000
H	-4.74390	-0.43436	0.00000
N	4.81910	-1.45873	0.00000
C	5.69470	-0.35440	0.00000
H	6.74832	-0.56741	0.00000
C	5.13133	0.91111	0.00000
H	5.76922	1.78357	0.00000
C	3.76411	1.06583	0.00000
N	3.08999	2.25228	0.00000
H	2.08374	2.29862	0.00000
H	3.60938	3.10792	0.00000
N	2.95944	-0.08537	0.00000
C	3.46664	-1.35441	0.00000
O	2.74193	-2.35145	0.00000
H	5.17107	-2.39944	0.00000

N_{Im}= 1 (248*i* cm^{-1})

WC alternative G*•C (C_s)

	X	Y	Z
N	-4.05067	-2.41356	0.00000
C	-4.19957	-3.76338	0.00000
H	-5.17513	-4.22724	0.00000
N	-3.05635	-4.43343	0.00000
C	-2.08952	-3.48207	0.00000
C	-0.66429	-3.58629	0.00000
O	0.04010	-4.59928	0.00000
N	-0.03649	-2.29920	0.00000
H	0.98347	-2.29157	0.00000
C	-0.71993	-1.11614	0.00000
N	-0.00626	0.00708	0.00000
H	0.99374	0.01051	0.00000
H	-0.51934	0.87238	0.00000
N	-2.06617	-1.00629	0.00000
C	-2.69134	-2.16783	0.00000
H	-4.78035	-1.72092	0.00000
N	4.79335	-0.97281	0.00000
C	5.59717	-2.08411	0.00000
H	6.66490	-1.91569	0.00000
C	5.02705	-3.31671	0.00000
H	5.63626	-4.20971	0.00000
C	3.60580	-3.38896	0.00000
N	2.96697	-4.57488	0.00000
H	1.94676	-4.61336	0.00000
H	3.48864	-5.43154	0.00000
N	2.83957	-2.28022	0.00000
C	3.40074	-1.06074	0.00000
O	2.75338	-0.00563	0.00000
H	5.17664	-0.04235	0.00000

N_{Im}= 4 (405*i*, 112*i*, 75*i*, and 61*i* cm^{-1})

WC alternative [G$\rightarrow$C]* (C_s)

	X	Y	Z
N	-3.76414	-2.29388	0.00000
C	-3.96935	-3.63896	0.00000
H	-4.96083	-4.06582	0.00000

N	-2.85324	-4.34058	0.00000
C	-1.87059	-3.41768	0.00000
C	-0.42066	-3.57579	0.00000
O	0.18204	-4.62671	0.00000
N	0.21093	-2.34572	0.00000
C	-0.39180	-1.09928	0.00000
N	0.37908	-0.06211	0.00000
H	-0.19228	0.77757	0.00000
N	-1.76156	-0.96538	0.00000
C	-2.39889	-2.10480	0.00000
H	-4.45988	-1.56622	0.00000
N	5.04870	-0.88484	0.00000
C	5.91290	-2.00154	0.00000
H	6.97023	-1.81195	0.00000
C	5.29501	-3.24220	0.00000
H	5.90100	-4.13862	0.00000
C	3.91964	-3.33808	0.00000
N	3.24110	-4.52780	0.00000
H	2.23745	-4.56727	0.00000
H	3.75010	-5.38994	0.00000
N	3.10021	-2.19183	0.00000
C	3.71171	-1.04483	0.00000
O	3.06214	0.10345	0.00000
H	5.39972	0.05580	0.00000
H	1.24292	-2.34011	0.00000
H	2.07868	-0.02526	0.00000

N_{Im}= 1 (281*i* cm^{-1})

WC G•C* (C_s)

	X	Y	Z
N	6.98515	-1.94172	0.00000
C	7.10630	-0.56728	0.00000
H	8.07272	-0.08522	0.00000
N	5.95897	0.04074	0.00000
C	5.02818	-0.97646	0.00000
C	3.59818	-0.93894	0.00000
O	2.84987	0.02808	0.00000
N	3.06031	-2.23857	0.00000
H	2.05136	-2.28594	0.00000
C	3.78066	-3.40107	0.00000
N	3.07426	-4.54991	0.00000
H	2.07208	-4.56968	0.00000
H	3.59666	-5.40707	0.00000
N	5.09395	-3.44547	0.00000
C	5.64897	-2.21776	0.00000
H	7.72391	-2.62465	0.00000
N	-1.89819	-3.61545	0.00000
C	-2.72629	-2.51324	0.00000
H	-3.79195	-2.66761	0.00000
C	-2.08376	-1.25127	0.00000
H	-2.69391	-0.35709	0.00000
C	-0.69964	-1.15780	0.00000
N	-0.00540	0.00219	0.00000
H	0.99460	0.00381	0.00000
H	-0.48948	0.88065	0.00000
N	0.09387	-2.31521	0.00000
C	-0.51988	-3.47835	0.00000
O	0.10926	-4.57332	0.00000
H	-2.25001	-4.55995	0.00000

N_{Im}= 4 (168*i*, 80*i*, 68*i*, and 11*i* cm^{-1})

WC [G→C]* (C_s)

	X	Y	Z
N	6.95821	2.16758	0.00000
C	7.18283	0.81848	0.00000
H	8.17877	0.40415	0.00000
N	6.06843	0.12333	0.00000
C	5.06441	1.06669	0.00000

C	3.66540	0.96347	0.00000
O	2.96291	-0.18954	0.00000
N	2.98215	2.20714	0.00000
H	1.90437	2.21670	0.00000
C	3.64247	3.41030	0.00000
N	2.85552	4.52095	0.00000
H	1.84721	4.49241	0.00000
H	3.32803	5.40521	0.00000
N	4.93572	3.54851	0.00000
C	5.60097	2.34463	0.00000
H	7.64346	2.90227	0.00000
H	2.02148	-0.02645	0.00000
N	-2.22905	3.31889	0.00000
C	-2.90645	2.14184	0.00000
H	-3.98644	2.20656	0.00000
C	-2.22790	0.97586	0.00000
H	-2.73779	0.02412	0.00000
C	-0.78846	1.05986	0.00000
N	0.02150	-0.01843	0.00000
H	-0.53228	-0.87545	0.00000
N	-0.13750	2.21831	0.00000
C	-0.83598	3.41258	0.00000
O	-0.29052	4.49253	0.00000
H	-2.72211	4.19983	0.00000

N_{Im}= 3 (404*i*, 167*i*, and 132*i* cm^{-1})

non-WC G•C (C_s)

	X	Y	Z
C	-0.40865	-1.29519	0.00000
N	0.01878	0.03411	0.00000
C	-0.78700	1.13362	0.00000
N	-2.09820	1.08159	0.00000
C	-2.55249	-0.18860	0.00000
C	-1.83397	-1.37777	0.00000
N	-2.68136	-2.46641	0.00000
C	-3.87270	-1.95159	0.00000
N	-3.86148	-0.57155	0.00000
N	-0.14853	2.32579	0.00000
O	0.42553	-2.19986	0.00000
H	1.04406	0.18903	0.00000
H	0.85720	2.37711	0.00000
H	-0.70214	3.16244	0.00000
H	-4.65268	0.04998	0.00000
H	-4.79792	-2.50866	0.00000
N	3.15462	-1.62491	0.00000
C	3.55669	-0.28955	0.00000
N	4.88239	-0.01833	0.00000
C	5.75120	-1.01126	0.00000
C	5.37379	-2.39058	0.00000
C	4.04191	-2.63833	0.00000
O	2.68944	0.59098	0.00000
N	7.06006	-0.67485	0.00000
H	2.13939	-1.83275	0.00000
H	3.62222	-3.63677	0.00000
H	6.10045	-3.19004	0.00000
H	7.78564	-1.36718	0.00000
H	7.30003	0.30175	0.00000

N_{Im}= 1 (71*i* cm^{-1})

non-WC G*•C (C_s)

	X	Y	Z
C	-0.68422	-1.27631	0.00000
N	-0.00429	-0.00706	0.00000
C	-0.65254	1.19539	0.00000
N	-1.98037	1.36349	0.00000
C	-2.65509	0.22118	0.00000
C	-2.09259	-1.12233	0.00000
N	-3.10045	-2.03538	0.00000
C	-4.21882	-1.32253	0.00000

N	-4.02620	0.01997	0.00000
N	0.14050	2.28266	0.00000
O	0.02546	-2.30283	0.00000
H	0.99571	-0.00938	0.00000
H	1.14532	2.18408	0.00000
H	-0.28423	3.19323	0.00000
H	-4.73362	0.73456	0.00000
H	-5.20975	-1.75326	0.00000
N	2.79223	-2.08609	0.00000
C	3.42522	-0.84034	0.00000
N	4.77940	-0.81021	0.00000
C	5.45855	-1.94108	0.00000
C	4.84330	-3.23101	0.00000
C	3.48734	-3.23911	0.00000
O	2.72876	0.17897	0.00000
N	6.80826	-1.84140	0.00000
H	1.75406	-2.12519	0.00000
H	2.89714	-4.14735	0.00000
H	5.41678	-4.14671	0.00000
H	7.39962	-2.65119	0.00000
H	7.21680	-0.92270	0.00000

N_{Im}= 4 (443*i*, 340*i*, 92*i*, and 67*i* cm^{-1})

non-WC [G→C]* (C_s)

	X	Y	Z
C	-0.71479	-1.17982	0.00000
N	0.00002	0.00006	0.00000
C	-0.59567	1.17202	0.00000
N	-1.96131	1.42931	0.00000
C	-2.65461	0.33291	0.00000
C	-2.16404	-1.00660	0.00000
N	-3.17725	-1.89083	0.00000
C	-4.27019	-1.14887	0.00000
N	-4.02539	0.19013	0.00000
N	0.15698	2.28136	0.00000
O	-0.16189	-2.26677	0.00000
H	1.16210	2.21052	0.00000
H	-0.29210	3.17963	0.00000
H	-4.70092	0.93654	0.00000
H	-5.27485	-1.54384	0.00000
N	2.95857	-2.01456	0.00000
C	3.55314	-0.79532	0.00000
N	4.82074	-0.58425	0.00000
C	5.63923	-1.71527	0.00000
C	5.13515	-2.99040	0.00000
C	3.74996	-3.18536	0.00000
O	2.73957	0.26523	0.00000
N	6.98005	-1.40330	0.00000
H	1.95741	-2.10221	0.00000
H	3.23731	-4.12787	0.00000
H	5.79499	-3.84810	0.00000
H	7.68585	-2.11236	0.00000
H	7.24010	-0.43619	0.00000
H	1.80002	0.00007	0.00000

N_{Im}= 1 (482*i* and 13*i* cm^{-1})

WC A*•T (C_1)

	X	Y	Z
O	-1.85163	2.13679	-0.12579
C	-2.64260	1.19814	-0.09802
N	-2.19055	-0.10195	-0.02050
C	-2.96198	-1.24032	0.03613
O	-2.51328	-2.36072	0.11855
N	-4.32373	-0.98644	-0.01006
C	-4.85729	0.27461	-0.09616
C	-4.09209	1.37955	-0.14292
N	0.97289	1.88488	0.14277
C	1.44222	0.64314	0.18331

N	0.61342	-0.39453	0.06856
C	1.19328	-1.62385	0.28749
N	2.46947	-1.84463	-0.25278
C	3.30695	-0.87296	-0.02351
C	2.86592	0.42536	0.33910
N	3.88049	1.32988	0.19426
C	4.92482	0.62942	-0.15631
N	4.63653	-0.70603	-0.34139
H	5.92561	1.01594	-0.27709
H	0.55181	-2.47786	0.45161
H	1.62187	2.64857	0.24920
H	-0.03384	2.05337	0.03516
H	-1.15265	-0.24538	0.00333
H	-5.93974	0.31960	-0.12288
H	5.27668	-1.43182	-0.61525
H	-4.91574	-1.79999	0.02954
C	-4.63485	2.77135	-0.23442
H	-4.30820	3.37066	0.61891
H	-4.26721	3.27169	-1.13361
H	-5.72646	2.76435	-0.25958

Sum of electronic and thermal free energy: –921.03406 a.u.

N_{Im}= 0

WC [A→T]* (C_1)

	X	Y	Z
O	-1.92895	-1.88714	0.75402
C	-2.56210	-0.81095	0.24595
N	-1.92757	0.43296	0.36486
C	-2.49498	1.62638	-0.01132
O	-1.90136	2.68879	0.06034
N	-3.78104	1.51806	-0.46845
C	-4.48967	0.30759	-0.55488
C	-3.87602	-0.86732	-0.18811
N	0.92320	-1.72515	0.29857
C	1.65856	-0.63002	0.30613
N	1.04141	0.54246	0.65009
C	1.71467	1.67104	0.63839
N	3.02148	1.85099	0.33180
C	3.62099	0.72561	0.01341
C	3.03715	-0.55258	-0.03146
N	3.94608	-1.49651	-0.40953
C	5.05475	-0.82091	-0.59041
N	4.92984	0.51733	-0.35312
H	5.99428	-1.25578	-0.89777
H	1.15974	2.56594	0.89387
H	1.50715	-2.51651	0.02445
H	-0.95711	0.48697	0.63988
H	-5.49549	0.36737	-0.93694
H	5.64447	1.22220	-0.43211
H	-4.21257	2.38648	-0.73022
C	-4.59714	-2.18360	-0.24650
H	-4.03894	-2.91022	-0.84028
H	-4.72111	-2.60768	0.75283
H	-5.58585	-2.05938	-0.69195
H	-0.97524	-1.82654	0.62313

Sum of electronic and thermal free energy: –921.06397 a.u.

N_{Im}= 0

WC G*•C (C_1)

	X	Y	Z
N	-0.74362	0.20970	0.67433
C	-1.34320	-0.90525	0.06414
C	-2.72438	-0.63350	-0.32704
C	-3.33888	0.59216	0.13540
N	-2.70285	1.62463	0.56432
C	-1.32604	1.38960	0.16742
N	-0.53988	2.48920	0.05580
O	-0.76719	-1.97282	-0.05322

N	-3.68252	-1.55340	-0.64614
C	-4.81925	-0.95355	-0.41218
N	-4.69250	0.31087	0.10785
H	-1.00980	3.33139	-0.23319
H	-5.43283	0.95650	0.32256
H	-5.79103	-1.38791	-0.60055
C	2.85900	1.08064	-0.23239
N	2.16568	-0.05662	0.02115
C	2.79022	-1.21847	0.16160
C	4.22526	-1.33130	0.10361
C	4.91351	-0.19456	-0.12636
N	4.25706	0.97955	-0.29350
O	2.34558	2.17666	-0.40947
N	2.03979	-2.30390	0.36856
H	4.75117	1.83862	-0.47704
H	5.99372	-0.16055	-0.19252
H	4.72812	-2.27856	0.23338
H	2.46630	-3.21096	0.42809
H	1.02011	-2.23338	0.28800
H	0.43391	2.38076	-0.23008
H	0.27956	0.17310	0.63655

Sum of electronic and thermal free energy: –937.10615 a.u.

N_{Im}= 0

WC [G→C]* (C_1)

	X	Y	Z
O	2.29853	2.20235	-0.07875
C	2.90130	1.12915	-0.05198
N	2.25601	-0.06870	0.06160
C	2.91565	-1.30577	0.11733
C	4.28893	-1.31682	0.02643
C	4.99098	-0.13334	-0.10945
N	4.25644	1.06804	-0.13500
N	2.10890	-2.39531	0.36608
O	-0.88195	-2.04765	-0.18148
C	-1.41080	-0.94928	-0.09313
N	-0.68534	0.21251	-0.01062
C	-1.27001	1.39324	0.07122
N	-2.63921	1.66063	0.11308
C	-3.34088	0.57237	0.04260
C	-2.86257	-0.76391	-0.06561
N	-0.51428	2.48870	0.12296
N	-3.88489	-1.63875	-0.11828
C	-4.97142	-0.89126	-0.04542
N	-4.71489	0.44123	0.05303
H	2.52792	-3.28634	0.16078
H	-0.97422	3.38010	0.18120
H	1.14207	-2.33261	0.07924
H	0.50716	2.42181	0.06486
H	4.82235	-2.25593	0.06937
H	6.05833	-0.05303	-0.21183
H	1.23955	-0.02501	0.07490
H	-5.97945	-1.27751	-0.06051
H	-5.38262	1.19131	0.11908
H	4.71819	1.95687	-0.21002

Sum of electronic and thermal free energy: –937.15689 a.u.

N_{Im}= 0
